# Common Genetic Determinants of Intraocular Pressure and Primary Open-Angle Glaucoma

**DOI:** 10.1371/journal.pgen.1002611

**Published:** 2012-05-03

**Authors:** Leonieke M. E. van Koolwijk, Wishal D. Ramdas, M. Kamran Ikram, Nomdo M. Jansonius, Francesca Pasutto, Pirro G. Hysi, Stuart Macgregor, Sarah F. Janssen, Alex W. Hewitt, Ananth C. Viswanathan, Jacoline B. ten Brink, S. Mohsen Hosseini, Najaf Amin, Dominiek D. G. Despriet, Jacqueline J. M. Willemse-Assink, Rogier Kramer, Fernando Rivadeneira, Maksim Struchalin, Yurii S. Aulchenko, Nicole Weisschuh, Matthias Zenkel, Christian Y. Mardin, Eugen Gramer, Ulrich Welge-Lüssen, Grant W. Montgomery, Francis Carbonaro, Terri L. Young, The DCCT/EDIC Research Group, Céline Bellenguez, Peter McGuffin, Paul J. Foster, Fotis Topouzis, Paul Mitchell, Jie Jin Wang, Tien Y. Wong, Monika A. Czudowska, Albert Hofman, Andre G. Uitterlinden, Roger C. W. Wolfs, Paulus T. V. M. de Jong, Ben A. Oostra, Andrew D. Paterson, Wellcome Trust Case Control Consortium 2, David A. Mackey, Arthur A. B. Bergen, André Reis, Christopher J. Hammond, Johannes R. Vingerling, Hans G. Lemij, Caroline C. W. Klaver, Cornelia M. van Duijn

**Affiliations:** 1 Glaucoma Service, The Rotterdam Eye Hospital, Rotterdam, The Netherlands; 2 Department of Epidemiology, The Erasmus University Medical Center, Rotterdam, The Netherlands; 3 Department of Ophthalmology, The Erasmus University Medical Center, Rotterdam, The Netherlands; 4 Department of Neurology, The Erasmus University Medical Center, Rotterdam, The Netherlands; 5 Department of Ophthalmology, University Medical Center Groningen, University of Groningen, Groningen, The Netherlands; 6 Institute of Human Genetics, University Erlangen-Nuremberg, Erlangen, Germany; 7 Department of Twin Research and Genetic Epidemiology, King's College London, London, United Kingdom; 8 Queensland Institute of Medical Research, Brisbane, Australia; 9 Department of Molecular Ophthalmogenetics, The Netherlands Institute for Neuroscience (NIN), Royal Netherlands Academy of Arts and Sciences (KNAW), Amsterdam, The Netherlands; 10 Centre for Eye Research Australia, University of Melbourne, Royal Victorian Eye and Ear Hospital, Melbourne, Australia; 11 NIHR Biomedical Research Centre for Ophthalmology, Moorfields Eye Hospital NHS Foundation Trust and UCL Institute of Ophthalmology, London, United Kingdom; 12 Program in Genetics and Genome Biology, Hospital for Sick Children, Toronto, Canada; 13 Department of Ophthalmology, Amphia Hospital, Breda, The Netherlands; 14 Department of Ophthalmology, Franciscus Hospital, Roosendaal, The Netherlands; 15 Department of Internal Medicine, The Erasmus University Medical Center, Rotterdam, The Netherlands; 16 Molecular Genetics Laboratory, University Eye Hospital, Tübingen, Germany; 17 Department of Ophthalmology, University of Erlangen-Nuremberg, Erlangen, Germany; 18 University Eye Hospital, Würzburg, Germany; 19 Department of Ophthalmology, Friedrich-Alexander University, Erlangen, Germany; 20 Center for Human Genetics, Duke University, Durham, North Carolina, United States of America; 21 Wellcome Trust Centre for Human Genetics, Oxford, United Kingdom; 22 MRC Social Genetic and Developmental Psychiatry Research Centre, Institute of Psychiatry, King's College, London, United Kingdom; 23 Department of Ophthalmology, School of Medicine, Aristotle University of Thessaloniki, AHEPA Hospital, Thessaloniki, Greece; 24 Centre for Vision Research, University of Sydney, Sydney, Australia; 25 Singapore National Eye Centre and Singapore Eye Research Institute, Singapore, Singapore; 26 Yong Loo Lin School of Medicine, National University of Singapore, Singapore, Singapore; 27 Department of Ophthalmology, Academic Medical Center, Amsterdam, The Netherlands; 28 Department of Clinical Genetics, The Erasmus University Medical Center, Rotterdam, The Netherlands; 29 Dalla Lana School of Public Health, University of Toronto, Toronto, Canada; 30 Lions Eye Institute, University of Western Australia, Centre for Ophthalmology and Visual Science, Perth, Australia; 31 Department of Clinical Genetics, Academic Medical Center, Amsterdam, the Netherlands; Georgia Institute of Technology, United States of America

## Abstract

Intraocular pressure (IOP) is a highly heritable risk factor for primary open-angle glaucoma and is the only target for current glaucoma therapy. The genetic factors which determine IOP are largely unknown. We performed a genome-wide association study for IOP in 11,972 participants from 4 independent population-based studies in The Netherlands. We replicated our findings in 7,482 participants from 4 additional cohorts from the UK, Australia, Canada, and the Wellcome Trust Case-Control Consortium 2/Blue Mountains Eye Study. IOP was significantly associated with rs11656696, located in *GAS7* at 17p13.1 (p = 1.4×10^−8^), and with rs7555523, located in *TMCO1* at 1q24.1 (p = 1.6×10^−8^). In a meta-analysis of 4 case-control studies (total N = 1,432 glaucoma cases), both variants also showed evidence for association with glaucoma (p = 2.4×10^−2^ for rs11656696 and p = 9.1×10^−4^ for rs7555523). *GAS7* and *TMCO1* are highly expressed in the ciliary body and trabecular meshwork as well as in the lamina cribrosa, optic nerve, and retina. Both genes functionally interact with known glaucoma disease genes. These data suggest that we have identified two clinically relevant genes involved in IOP regulation.

## Author Summary

Glaucoma is a major eye disease in the elderly and is the second leading cause of blindness worldwide. The numerous familial glaucoma cases, as well as evidence from epidemiological and twin studies, strongly support a genetic component in developing glaucoma. However, it has proven difficult to identify the specific genes involved. Intraocular pressure (IOP) is the major risk factor for glaucoma and the only target for the current glaucoma therapy. IOP has been shown to be highly heritable. We investigated the role of common genetic variants in IOP by performing a genome-wide association study. Discovery analyses in 11,972 participants and subsequent replication analyses in a further 7,482 participants yielded two common genetic variants that were associated with IOP. The first (rs11656696) is located in *GAS7* at chromosome 17, the second (rs7555523) in *TMCO1* at chromosome 1. Both variants were associated with glaucoma in a meta-analysis of 4 case-control studies. *GAS7* and *TMCO1* are expressed in the ocular tissues that are involved in glaucoma. Both genes functionally interact with the known glaucoma disease genes. These data suggest that we have identified two genes involved in IOP regulation and glaucomatous neuropathy.

## Introduction

Primary open-angle glaucoma (hereafter referred to as glaucoma) is a progressive optic neuropathy responsible for 12.3% of global blindness [1]. The evidence for a genetic etiology of glaucoma is well-established [2]. However, genes consistently implicated so far (*MYOC*, *OPTN*, *WDR36*) [3]–[5] are relevant only in a limited number of families and explain a small proportion of the glaucoma cases in the general population [6]–[8]. So far, 3 genome-wide association studies (GWASs) for glaucoma have been published. A study from Iceland identified a common variant near *CAV1* and *CAV2* [9]. Both genes are expressed in the trabecular meshwork as well as in retinal ganglion cells. A Japanese study identified 3 putative loci, although none of these reached genome-wide significance [10]. A recent study in an Australian cohort of 590 patients with severe glaucomatous visual field loss identified susceptibility loci at *TMCO1* and *CDKN2B-AS1* [11]. The latter region had already at a genome-wide significant level been associated with vertical cup-to-disc ratio, which is an important clinical marker of glaucoma [12]. Finally, a study in an Afro-Caribbean population identified a genome-wide significant association between glaucoma and a locus on chromosome 2p by genotyping a previously identified linkage region [13].

Intraocular pressure (IOP) is the major risk factor of glaucoma and existing glaucoma therapies are exclusively aimed at lowering IOP. An elevated IOP (>21 mmHg) influences both the onset and the progression of glaucoma [14]. Genetic effects have been shown to account for a significant proportion of the variance in IOP, with heritability estimates ranging from 0.29 to 0.67 [15]–[19]. Five genome-wide linkage studies of IOP have been performed [20]–[24]. This resulted in 15 potential regions of interest, 2 of which were genome-wide significantly linked to IOP. The first was identified in an Australian glaucoma pedigree and was located on 10q22 [20]. The second was identified in individuals without glaucoma in West Africa and Mongolia and was located in the 5q22-23 region, which had already been implicated in glaucoma (*WDR36* gene and GLC1M locus) [3], [23]–[25]. Taken together, these findings suggest that extensive heterogeneity underlies the genetics of IOP and that the same genetic factors may possibly affect both the variance in normal IOP and the risk and onset of glaucoma. Thus, unraveling the genetic background of IOP may shed light upon the pathophysiology of glaucoma. To date, no GWAS has been reported for IOP.

To identify genetic determinants of IOP, we performed a GWAS in 11,972 participants from 4 independent population-based studies in The Netherlands, and we replicated our findings in 7,482 participants from 4 additional independent cohorts of Caucasian ancestry. We investigated whether the IOP associated SNPs were also related to glaucoma in 1,432 glaucoma cases. Lastly, we examined expression levels of the identified candidate genes in human ocular tissues. We identified common variants in *GAS7* and *TMCO1* that altered the susceptibility to both IOP and glaucoma.

## Results

### Discovery studies

Genotypic and IOP data were available for 11,972 participants from the Rotterdam Study cohort I (RS-I), RS-II, RS-III, and the Erasmus Rucphen Family (ERF) Study (Table 1). Genomic inflation factors were 1.037 for RS-I, 1.006 for RS-II, 1.015 for RS-III, and 1.029 for ERF. QQ-plots for the observed versus expected p-values for the individuals cohorts as well as for the discovery meta-analysis have been provided in Figure S1. The genome-wide association analyses in the ERF study were performed with and without adjustment for the time of the IOP measurement. As this adjustment did not significantly affect the results, the unadjusted (other than for age and sex) data were taken forward to the meta-analysis.

**Table 1:** Characteristics of the discovery cohorts.

Characteristic	RS-I	RS-II	RS-III	ERF
Participants with valid data (N)	5,794	2,102	2,041	2,035
Age (y), mean ± SD (range)	68.8±8.9 (55–100)	64.4±8.0 (55–95)	55.7±5.8 (45–97)	48.8±14.4 (18–86)
Male gender (%)	41.2	45.7	43.9	43.3
IOP (mmHg), mean ± SD (range)	14.7±3.4 (5–59)	14.4±3.4 (7–32)	13.6±3.0 (5–30)	15.3±3.1 (6–33)
IOP≥22 mmHg (%)	3.3	3.3	1.9	1.2
Participants with IOP lowering treatment (%)	2.4	3.9	1.5	0.9
Vertical cup-disc ratio, mean ± SD (range)	0.50±0.14 (0.00–0.89)	0.50±0.14 (0.05–0.87)	0.42±0.17 (0.00–1.00)	0.43±0.16 (0.00–0.83)
Disc area (mm2), mean ± SD (range)	2.42±0.48 (0.58–5.44)	2.32±0.48 (1.06–6.20)	1.92±0.45 (0.70–7.20)	1.90±0.35 (1.07–3.95)

IOP = intraocular pressure; SD = standard deviation; RS = Rotterdam Study; ERF = Erasmus Rucphen Family study.

**Figure 1:**
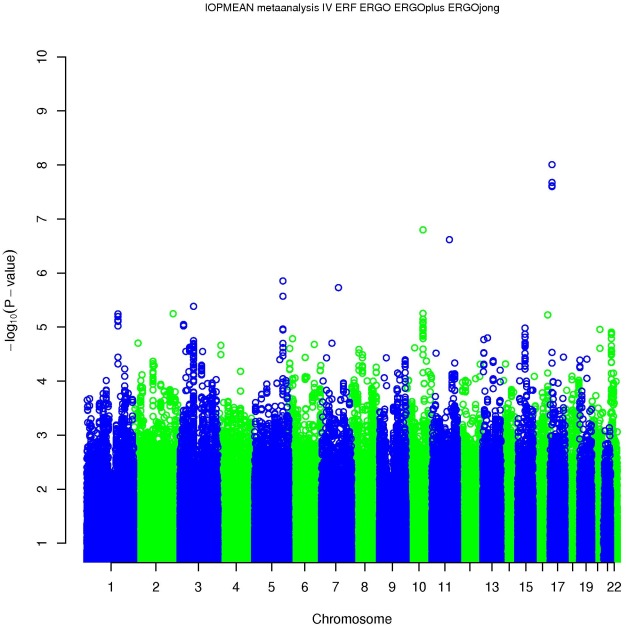
Results of the meta-analysis of the gene discovery cohorts.

**Figure 2:**
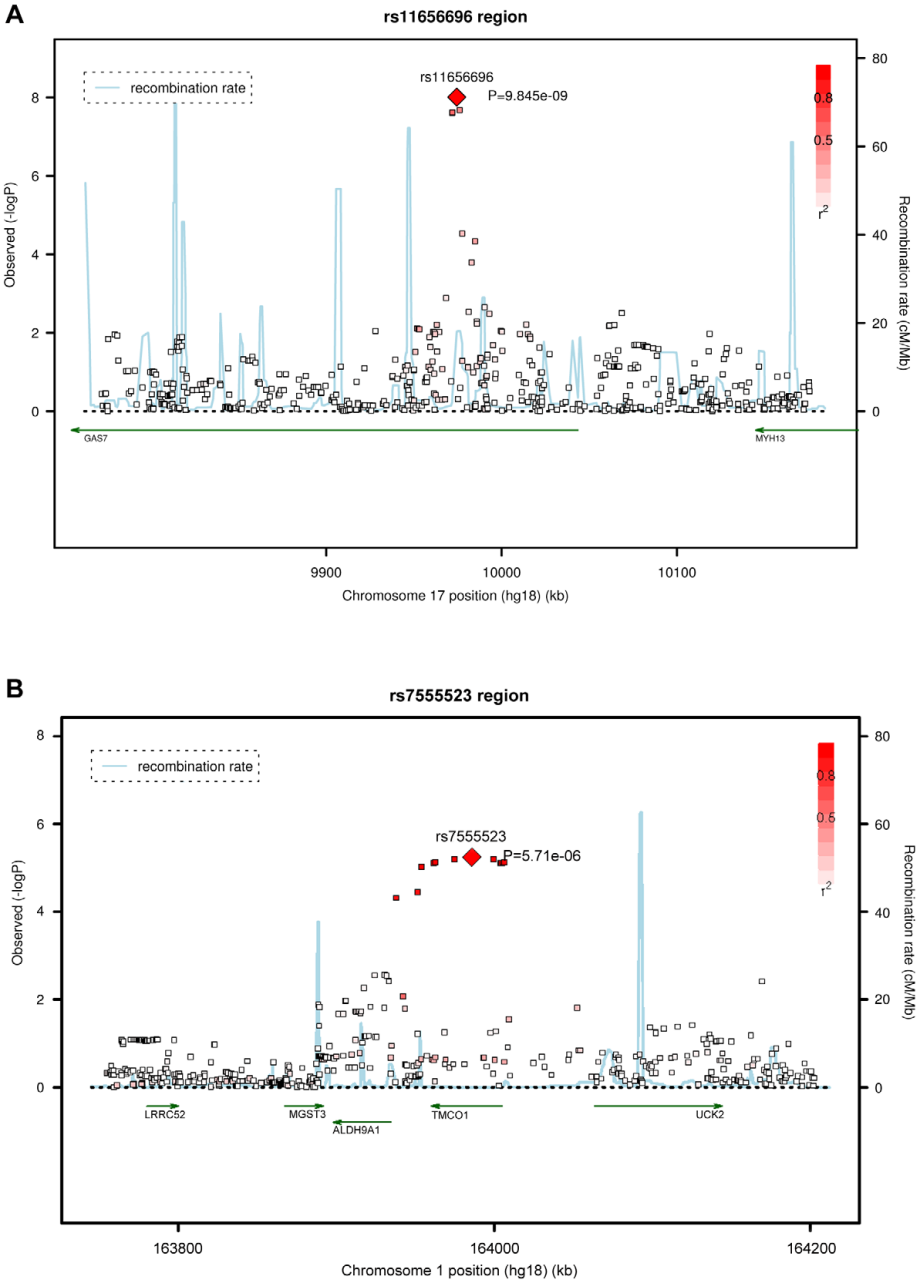
Regional association plots of the 17p13.1 and 1q24.1 regions in the discovery meta-analysis.

Four SNPs on chromosome 17p13.1 were significantly associated with IOP in the discovery meta-analysis (p<5×10^−8^; Figure 1, Table 2). These SNPs are located in the growth arrest-specific 7 (*GAS7*) gene (Figure 2) [26]. The SNP that showed strongest association with IOP was rs11656696. The effect of the rs11656696 alleles was consistent across all 4 discovery cohorts (Table S1). A further 6 chromosomal loci showed more moderate but nevertheless suggestive associations with IOP (p<1×10^−5^; Table 2, Figure S2) and were also taken to the replication phase. Of these, rs7555523 is located in the trans-membrane and coiled-coil domains 1 (*TMCO1*) gene on chromosome 1q24.1 (Figure 2) [26], which is located 7.6 MB from *MYOC*. A list of all the SNPs that were associated with IOP at a significance level of p<1×10^−5^ has been provided in Table S2.

**Table 2:** Results of the meta-analysis of the gene discovery cohorts: loci associated with IOP (p<10^−5^).

SNP	Chrom	Position	MA	MAF	Gene region	#SNPs*	Beta	SE	P-value
rs11656696	17p13.1	9974404	A	0.43	*GAS7*	4	−0.26	0.05	9.8E-09
rs7894966	10q23.2	88608604	G	0.04	*BMPR1A*	8	0.67	0.13	1.6E-07
rs216146	5q32	149426114	T	0.39	*CSF1R*	2	0.22	0.05	1.4E-06
rs2117760	3p13	70933151	A	0.32	*FOXP1*	1	0.22	0.05	4.1E-06
rs7555523	1q24.1	163985603	C	0.12	*TMCO1*	11	0.30	0.07	5.7E-06
rs1826598	16q23.1	76130456	A	0.11	*ADAMTS18, NUDT7*	1	0.32	0.07	6.0E-06
rs9841621	3p24.3	18384081	G	0.01	*SATB1*	5	−0.81	0.18	8.9E-06

SNP = single nucleotide polymorphism; Chrom = chromosome; MA(F) = minor allele (frequency); SE = standard error.

*: number of SNPs with p<10^−5^ in the region.

According NCBI build 37.1, rs11656696 is located at position 10033679 in the growth-arrest-specific gene *GAS7* while an earlier build allocated the SNP at 9974404 (http://www.ncbi.nlm.nih.gov).

We examined at least 416 KB of the chromosomal regions spanning the known disease genes *MYOC*, *OPTN*, and *WDR36* in more detail in the discovery meta-analysis. None of the 1507 SNPs assessed in total showed significant association with IOP (Figure S3) [26]. We also evaluated 12 SNPs which had approached genome-wide significance in earlier association studies (Table S3) [9], [10], [13]. Of these, rs4236601 in the *CAV1-CAV2* region, previously identified in Caucasians, was consistently associated with increased IOP in our discovery meta-analysis (beta = 0.19, 95%CI = 0.09–0.29, p = 1.1×10^−4^) [9]. The rs4656461 locus, identified in patients with severe glaucoma from Australia, overlapped with the rs7555523 locus that was identified with suggestive evidence in our study [11]. The 2 SNPs are at a disctance of 31774 base pairs from each other and are in linkage disequilibrium (R squared = 1). Rs4977756, the second locus that emerged from the study in Australia, was not associated with IOP in our discovery cohorts. Of the three regions identified in Japan, only rs7081455 on chromosome 10 showed nominal evidence for association with IOP (beta = 0.12, 95%CI = 0.08–0.16, p = 4.6×10^−3^). Our data did not replicate the association in the 2p16 locus which was previously identified in Afro-Caribbeans. Finally, we examined the two chromosomal regions that had previously been identified in genome-wide linkage studies of IOP [20], [24]. Both regions showed suggestive evidence of association with IOP in our discovery meta-analysis: Rs7894966, located in the bone morphogenetic protein receptor 1A (*BMPR1A*) gene on chromosome 10q23.2, is in the region previously identified in an Australian linkage study of IOP (16.2 MB from the peak LOD score) [20]; Rs216146, in the colony stimulating factor 1 receptor (*CSF1R*) gene on chromosome 5q32, is close to the region that previously showed genome-wide significant linkage to IOP in West Africans [24]. This SNP is located at a distance of 21.0 MB to the peak LOD score, 10.0 MB to the glaucoma locus *GLC1M*, and 39.0 MB to *WDR36*.

### Replication studies

Replication of the IOP association was done in 4 additional cohorts from the TwinsUK study (N = 2,235), the Australian Twin study (N = 1,807), the Diabetes Control and Complications Trial/Epidemiology of Diabetes Interventions and Complications study (DCCT/EDIC; N = 1,304), and the Wellcome Trust Case-Control Consortium 2 / Blue Mountains Eye Study (WTCCC2/BMES; N = 2,136) (Text S1). The results of the replication analyses are presented in Table 3. Although in most studies the association did not reach nominal significance (p<0.05), most likely explained by the low statistical power of these relatively small studies, the directionality of the effects was consistent across the 4 replication cohorts for most SNPs. The exceptions were rs7894966 and rs216146 for which the effects were in opposite direction compared to the discovery cohorts. When the gene discovery and replication cohorts were combined, two intronic SNPs reached genome-wide significance. Each copy of the rs11656696 minor allele (A), located in *GAS7*, was associated with a 0.19 mmHg IOP reduction (95% confidence interval [CI] = 0.12–0.26 mmHg; p = 1.4×10^−8^), and each copy of the rs7555523 minor allele (C), located in *TMCO1*, with a 0.28 mmHg IOP increase (95%CI = 0.18–0.37 mmHg; p = 1.6×10^−8^).

**Table 3:** Results of the replication analyses and the joint analysis of discovery and replication cohorts.

	Replication analyses																				Joint analysis of discovery and replication cohorts		
	TWINS-UK					Australian Twins					DCCT/EDIC					WTCCC2/BMES							
SNP	MAF	Beta	SE	P-value	*	MAF	Beta	SE	P-value	*	MAF	Beta	SE	P-value	*	MAF	Beta	SE	P-value	*	Beta	SE	P-value
rs11656696	0.42	−0.32	0.11	3.9^E^-03	P	0.42	−0.11	0.10	2.9E-01	G	0.42	0.04	0.11	6.8E-01	G	0.42	−0.06	0.09	4.9E-01	G	**−0.19**	**0.03**	**1.4E-08**
rs7894966	0.02	−1.15	0.37	1.7^E^-03	I	0.03	−0.32	0.29	2.6E-01	I	0.04	−0.11	0.29	6.9E-01	I						**0.30**	**0.10**	**3.6E-03**
rs216146	0.39	0.00	0.11	9.8^E^-01	I	0.37	−0.08	0.11	4.8E-01	I	0.43	−0.08	0.11	4.7E-01	G	0.41	−0.08	0.09	3.4E-01	I	**0.09**	**0.03**	**4.9E-03**
rs2117760	0.30	0.12	0.11	2.9^E^-01	I	0.30	−0.03	0.11	7.7E-01	I	0.33	0.05	0.11	6.5E-01	I						**0.16**	**0.04**	**2.8E-05**
rs7555523	0.12	0.24	0.15	9.6^E^-02	I	0.12	0.23	0.16	1.4E-01	I	0.11	0.18	0.17	2.9E-01	G	0.12	0.30	0.13	1.8E-02	I	**0.28**	**0.05**	**1.6E-08**
rs1826598	0.11	0.15	0.15	3.4^E^-01	P	0.10	0.10	0.17	5.6E-01	G	0.11	0.20	0.18	2.7E-01	G	0.12	0.01	0.13	9.3E-01	G	**0.22**	**0.05**	**2.0E-05**
rs9841621	0.02	−0.42	0.36	2.4^E^-01	P	0.02	−0.34	0.33	3.0E-01	I	0.02	−0.31	0.40	4.3E-01	G	0.02	−0.15	0.31	6.3E-01	G	**−0.54**	**0.12**	**1.4E-05**

DCCT/EDIC = Diabetes Control and Complications Trial / Epidemiology of Diabetes Interventions and Complications study; WTCCC2/BMES = Wellcome Trust Case-Control Consortium 2 / Blue Mountains Eye Study; SNP = single nucleotide polymorphism; MAF = minor allele frequency; SE = standard error;

*: Column indicates whether the SNP has been genotyped (G), imputed (I), or partly (P) genotyped, i.e. genotyped in 2/3 of the participants.

### Glaucoma case-control studies

**Figure 3:**
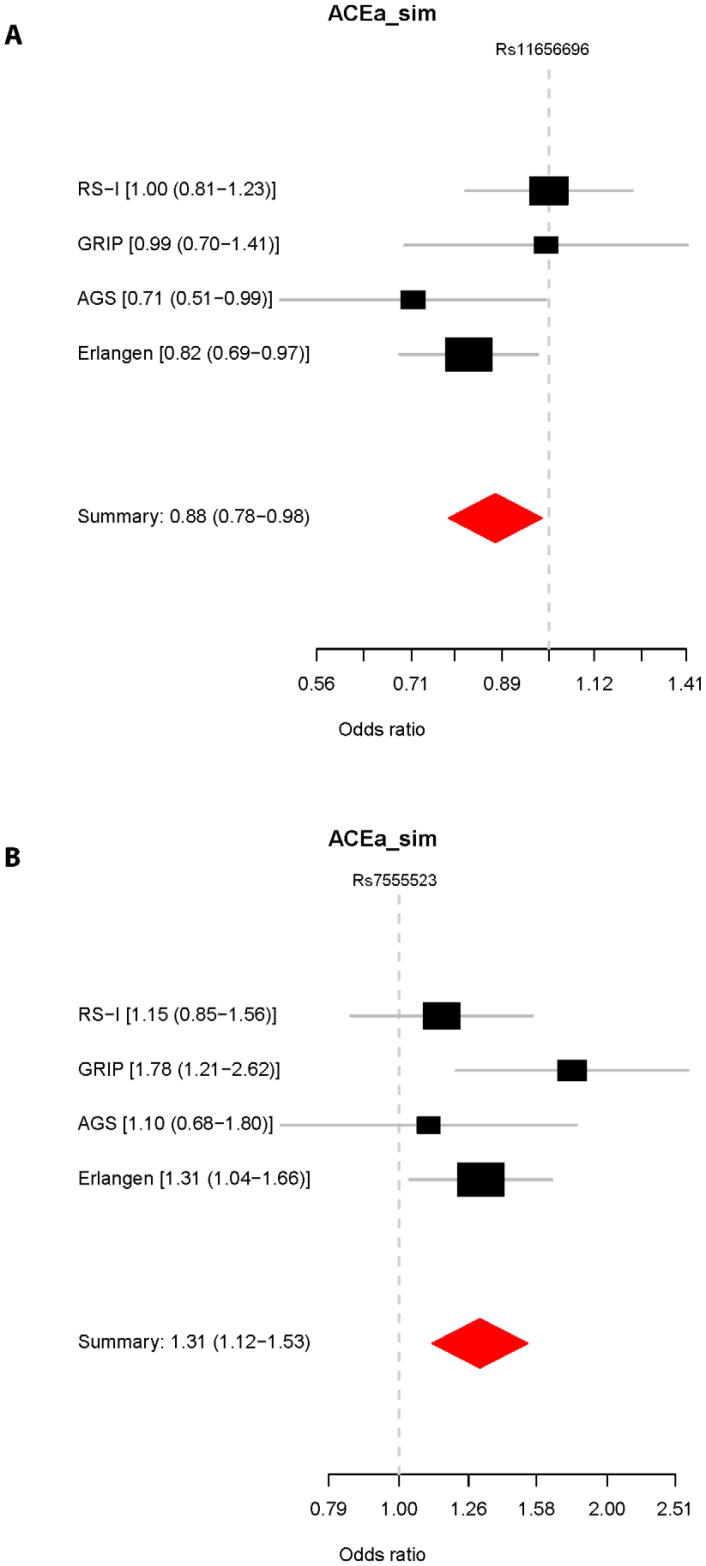
Association of rs11656696 and rs7555523 with glaucoma.

We investigated the associations of the *GAS7* rs11656696 minor allele (A) and the *TMCO1* rs7555523 minor allele (C) with glaucoma in 4 case-control studies from the Netherlands and Germany (Text S1). The results are presented in Figure 3. For rs11656696 A we found a decreased glaucoma risk in the Amsterdam Glaucoma Study (AGS; OR = 0.71, 95%CI = 0.51–0.99) and the Erlangen and Tübingen study (OR = 0.82, 95%CI = 0.69–0.97), but not in RS-I and the Genetic Research in Isolated Populations (GRIP) program. When combining the 4 case-control studies, rs11656696 A showed a decreased glaucoma risk (OR = 0.88, 95%CI = 0.78–0.98, p = 2.4×10^−2^). For rs7555523 C, we found an increased glaucoma risk in all 4 case-control studies. Combined, these studies showed an increased glaucoma risk with an OR of 1.31 (95%CI = 1.12–1.53, p = 9.1×10^−4^).

### Expression studies

**Figure 4:**
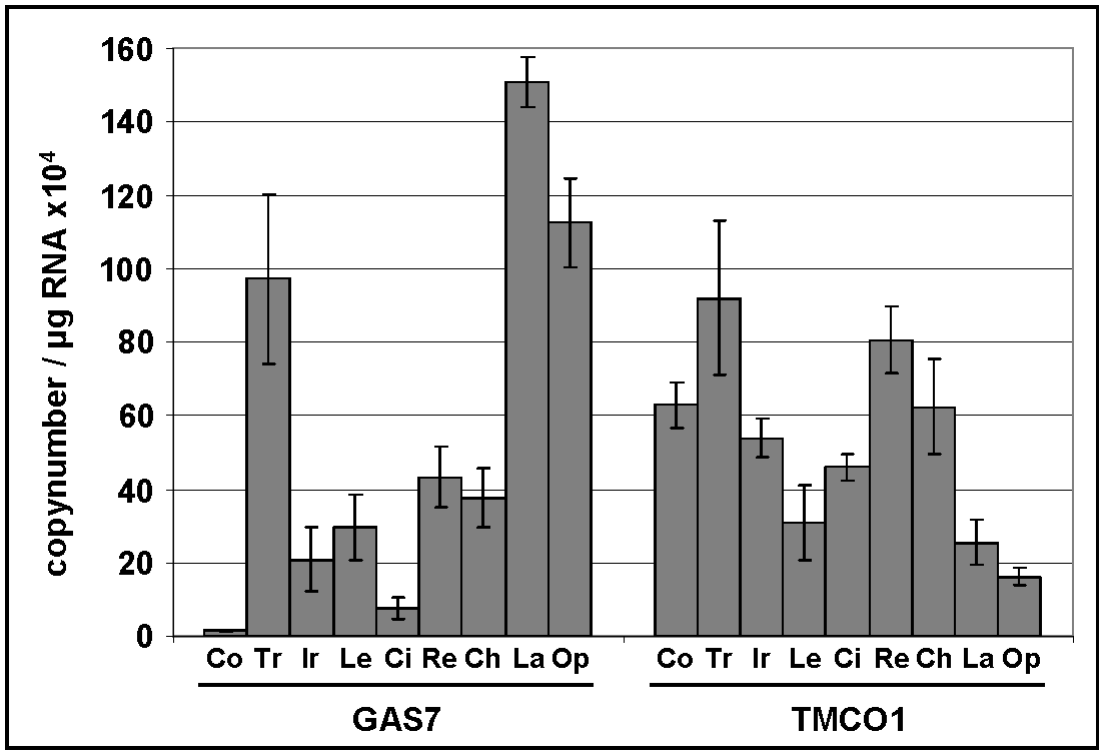
Expression levels of GAS7 and TMCO1 in human ocular tissues. Quantitative determination of *GAS7* and *TMCO1* mRNA expression levels in human ocular tissues by using real-time PCR technology (n = 4). The expression levels were normalized against GAPDH and the results are expressed as copynumber/µg RNA. Co, cornea; Tr, trabecular meshwork; Ir, iris; Le, lens; Ci, ciliary body; Re, retina; Ch, choroid; La, lamina cribrosa; Op, optic nerve.

In a first study of expression levels in human ocular tissues, we observed moderate to high expression of *GAS7*, and high expression of *TMCO1* in the ciliary body (CB), the secretory neuroepithelium that produces the aqueous humor (Table 4). Both genes were moderately to highly expressed in the choroid, the retinal pigment epithelium and photoreceptors. In a second, independent study, mRNA expression profiles in human eyes of *GAS7* and *TMCO1* displayed an ubiquitous expression of both gene products, with the highest expression levels of *GAS7* in the trabecular meshwork, the lamina cribrosa, and the optic nerve, whereas *TMCO1* expression was most prominent in the trabecular meshwork and the retina (Figure 4).

**Table 4:** Gene expression levels in human ocular tissues.

Gene	CB-PE	CB-NPE	Choroid	RPE	Photoreceptors	TM*
*GAS7*	55 (1.3)	57 (2.0)	73 (5.1)	76 (1.7)	78 (8.6)	78 (3.1)
*TMCO1*	93 (1.5)	93 (1.0)	86 (2.5)	88 (1.9)	88 (2.4)	88 (1.5)

The two genes are ranked by increasing expression, calculated by the mean percentiles (SD) of the expression levels. Gene expression of CB-PE and CB-NPE (n = 4), choroid (n = 3), photoreceptors (n = 3) and RPE (n = 6) were performed on Agilent Human 44k microarray of post-mortem donor eyes without glaucoma or any other ocular diseases.

*: Data from Liton et al., performed on Affymetrix Human U133 microarray, showing mean percentiles (SD) of human gene expression levels in TM tissue from 3 healthy eyes [28].

CB-PE = ciliary body, pigmented epithelium; CB-NPE = ciliary body, non-pigmented epithelium; RPE = retinal pigment epithelium; TM = trabecular meshwork.

## Discussion

We identified rs11656696 in *GAS7* and rs7555523 in *TMCO1* as common variants associated with IOP. In a joint analysis of the discovery and replication cohorts each copy of the rs11656696 minor allele (A; allele frequency 0.43) was associated with a 0.19 mmHg decrease in IOP (95%CI = 0.12–0.26 mmHg), whereas each copy of the rs7555523 minor allele (C; allele frequency 0.12) was associated with a 0.28 mmHg increase in IOP (95%CI = 0.18–0.37 mmHg). Both variants showed marginal evidence for association with glaucoma when combining data from 4 case-control studies, although for rs11656696 significance was only obtained in 2 studies.

**Figure 5:**
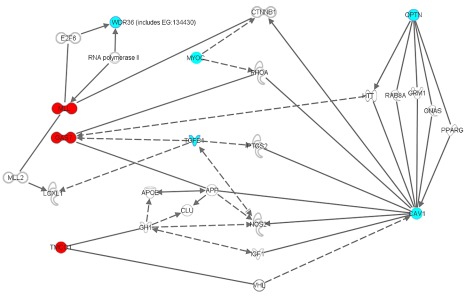
Biochemical and functional interactions between (putative) glaucoma disease genes. Ingenuity diagram of biochemical and functional interactions between the newly identified GAS7 and TMCO1 disease genes implicated in elevated IOP and glaucoma, and previously known glaucoma disease genes (WDR36, MYOC, OPTN, CAV1). Functional relationships in the knowledge database Ingenuity (www.ingenuity.com) are a compilation of all known gene-relevant biochemical and functional data of in vivo and in vitro experiments involving (molecules, cells and tissues of) rats and mice and man, as well as data from zebrafish and Drosophila and ongoing clinical trials in man. The query genes/proteins GAS7 (including it's drosophila homologue MLL) and TMCO1 are presented in red. Known glaucoma disease genes are given in blue. Blank genes/molecules are generated by the knowledge database to construct a functional network under the criteria specified by the investigator. The diagram was generated using the function “Path Explorer”. In general, solid lines indicate a direct, experimentally verified, physical relationship between two molecules, for example a physical protein-protein interaction, or an enzym-DNA interaction, etc. Dotted lines refer to the existence of an indirect functional relationship, such as co-upregulation in cell cultures under specific experimental conditions. WDR36 = WD Repeat-containing protein 36; OPTN = optineurin; MYOC = myocilin; GAS7 = growth arrest-specific 7; MLL = myeloid/lymphoid or mixed-lineage leukemia; TMCO1 = transmembrane and coiled-coil domains 1; CAV1 = caveolin 1; TGFB1 = transforming growth factor beta 1; CTNNB1 = catenin (cadherin-associated protein) beta 1; RHOA = ras homolog gene family, member A; E2F6 = E2F transcription factor 6; VHL = von Hippel-Lindau; HTT = huntingtin; NOS2 = nitric oxide synthase 2; LOXL1 = lysyl oxidase-like 1; APOE = apolipoprotein E; APP = amyloid beta (A4) precursor protein; CLU = clusterin. As shown, GAS7 (MLL) and TMCO1 interact multiple times and in several ways with previously known glaucoma disease genes. For a specific description of these interactions, see text.

*GAS7* is located in a chromosomal region previously identified by a linkage study of glaucoma [27]. We observed high expression levels of *GAS7* in the optic nerve, and, in particular, the lamina cribrosa. The lamina cribrosa is the connective tissue network through which the nerve fibers traverse to form the optic nerve, and is assumed to be the main site for glaucomatous damage to the optic nerve. We also observed moderate to high expression of *GAS7* in the ciliary body (CB), the secretory neuroepithelium that produces the aqueous humor, and high expression of *GAS7* in the trabecular meshwork (TM), which is the main tissue involved in aqueous humor outflow [28]. Together, the CB and TM largely control IOP. Previously, Liton and colleagues already reported significant downregulation of *GAS7* expression in TM of glaucomatous eyes [28]. In absence of the (in vivo) typical mechanical forces on the TM, a similar effect was also observed in cultured TM cells [28]. High *GAS7* expression has previously been shown in amacrine cells in the mouse retina, while lower expression was found in retinal cell types which are usually not affected by glaucoma [29]. Protein pathway analyses and evidence from previous literature allude to functional effects of *GAS7* in both the TM and retina. *GAS7* has been implicated in cell remodelling, possibly facilitated through its capacity to associate with actin and mediate the reorganization of microfilaments [30], [31]. In neuronal cells, GAS7 expression is critical for neurite formation [30], [32]. *MYOC*, the major glaucoma gene previously associated with elevated IOP cases, also affects the actin cytoskeletal structure and neurite outgrowth [33]. Whereas *MYOC* has an inhibitory effect on neurite outgrowth, *GAS7* is involved in the formation of neurites. Interestingly, experimental ischemic retinal damage in rats, resembling retinal damage due to glaucoma, leads to extensive remodelling of inner retinal neurons [34]. *GAS7* may also contribute to remodelling of the TM, as is the case for the myocilin protein which has been shown to alter the actin structure and modulate TM cell morphogenesis [35]. *GAS7* interacts with *MYOC*, as well as with other genes implicated in glaucoma, such as *OPTN*, *WDR36*, *CAV1*, *NOS2*, *FOXC1*, *APOE*, *APP* and *CLU* (Figure 5; www.ingenuity.com). The latter three genes are primarily known for their association with Alzheimer's Disease, a neurodegenerative disease previously linked to glaucoma [36]. *GAS7* interacts with both *MYOC* and *CAV1* through β-catenin (*CTNNB1*) and RhoA (*RHOA*). B-catenin anchors the actin cytoskeleton and is part of the Wnt signalling pathway, which has previously been implicated in trabecular outflow regulation [37], [38]. RhoA signalling regulates the intracellular levels of phosphorylated myosin light chain, which directly influence trabecular meshwork cellular contraction and thus aqueous humor outflow [39]. Finally, *GAS7* is regulated by transforming growth factor (TGF) beta, which has previously been implicated in trabecular outflow as well as in the development of the optic disc (the primary site of neuronal damage by glaucoma) [40]–[42]. The frequency of the *GAS7* rs11656696 A-allele is 0.44 in the HapMap CEU population of European ancestry whereas it is 0.12 in the HapMap Yorubian population of African ancestry. The lower frequency of the A-allele in the African population is consistent with the higher prevalence of glaucoma with elevated IOP in this population and warrants further research into the association of rs11656696 with IOP and glaucoma in African populations.

The second variant that we found to be associated with IOP and glaucoma was rs7555523 in *TMCO1*, a highly evolutionary conserved gene of largely unknown function [43], [44]. *TMCO1* has recently been associated with severe glaucomatous visual field loss, indicating that this locus may influence both the normal variance in IOP and the risk of developing severe glaucoma [11]. These findings support the hypothesis that studies of IOP can assist in identifying susceptibility genes for glaucoma. Rs7555523 is located in a region which previously showed suggestive evidence for linkage with blood pressure [45]. IOP and blood pressure have already been shown to correlate [46]. *TMCO1* is highly expressed in the human TM and CB, which together regulate IOP, and in the retina [28]. *TMCO1* interacts with *CAV1* via VHL (Figure 5). A homozygous frameshift mutation in *TMCO1* has been associated with a genetic syndrome involving multiple organ systems, including renal agenesis and hydronephrosis [43]. Extensive ophthalmic examination was not reported, however a high incidence of strabismus was noticed.

No previous GWASs of IOP have been conducted to date. When comparing our findings to those of association studies of glaucoma, we found an overlap with 3 regions. First, we replicated the association with the *TMCO1* region, as has been described in the previous paragraph. Second, rs4236601 in the *CAV1-CAV2* region, previously identified in Caucasians, was consistently associated with increased IOP in our discovery meta-analysis [9]. Our findings in this region did not reach genome-wide significance. However, multiple testing adjustment by using a Bonferroni correction for the 12 SNPs evaluated (Table S3) yields a criterion for significance of p<4×10^−3^. Thus, our findings strongly support an association between the *CAV1-CAV2* region and IOP, despite the fact that the original report that identified *CAV1-CAV2* did not find evidence for a stronger relation to high pressure glaucoma. Third, a locus on chromosome 10p, which had previously been identified in Japan, also passed this Bonferroni threshold [10]. Similar to Nakano and coworkers, we could not assign a specific glaucoma disease gene to this region. The replication of this locus in our study is remarkable as most glaucoma patients in Japan present with normal tension glaucoma (i.e., glaucoma with IOP≤21 mmHg).

Our study design had three potential limitations. First, we did not measure central corneal thickness (CCT) in the majority of the participants of the discovery cohorts. CCT is an important determinant of IOP measurements and may be an IOP-independent risk factor for glaucoma [47], [48]. The genes involved in CCT may also associate with IOP and glaucoma. CCT has previously been reported to account for 1–6% of the variance in IOP measured with Goldmann applanation tonometry [49]–[52]. Heritability estimates of CCT range from 0.68 to 0.95 [53]–[55], implying that this trait is even more heritable than IOP. Because we did not include CCT as a covariate in our discovery analyses, the identified SNPs may determine CCT rather than IOP. To test this hypothesis, we assessed whether the identified SNPs were associated with CCT in a randomly selected subpopulation of 784 participants from RS-I for whom CCT data were available. None of the 7 SNPs identified in the discovery meta-analysis was associated with CCT (p>0.24). The results for rs11656696 (p = 0.28) and for rs7555523 (p = 0.31) suggest that the associations of these SNPs with IOP were not explained by CCT. Furthermore, in a recent GWAS conducted in the Australian twins and TwinsUK cohorts, these SNPs were not associated with CCT [56]. We also assessed the associations of rs11656696 and rs75555623 with IOP in the TwinsUK cohort after including CCT as a covariate in the multivariate model. The association changed from −0.316 (95%CI = −0.536–−0.096) to −0.400 (95%CI = −0.620–−0.180) for rs11656696 and from 0.242 (95%CI = −0.048–0.532) to 0.220 (95%CI = −0.080–0.520) for rs7555523 after correction for CCT, suggesting that the controlling for CCT only produces relatively minor changes with respect to effect size and significance of association.

Second, in the gene discovery analyses, the initial IOP levels were not known for the participants who received IOP lowering medication or who had a history of IOP lowering surgery. We imputed these IOPs, because (particularly in the elderly population of RS-I) participants with extreme IOPs, which are likely to be genetically determined, are otherwise excluded. Similar approaches have been applied to research of blood pressure, where an analogous problem occurs: those with the higher blood pressures are otherwise excluded [57], [58]. Although the imputations for IOP lowering medication are based on a large meta-analysis [59], the justification for the imputations for IOP lowering surgery is not based on empirical evidence. Exclusion of any participants who received IOP lowering treatment or who had received this treatment in the past (either by medication or surgically), did not substantially change the betas for rs11656696 and rs7555523 (see Text S1). However, for rs11656696, it did result in a loss of statistical power, as the participants on treatment had a significantly (p = 8.1×10^−6^) lower frequency of the protective A-allele (odds ratio = 0.58, 95%CI = 0.45–0.74).

Third, some replication cohorts differed from the discovery cohorts with respect to their age, sex or disease status. The participants of the Australian Twin study and DCCT/EDIC were evidently younger than the participants of the other cohorts were. Aging has previously been associated with an increase in the accumulation of extracellular material in the trabecular meshwork, as well as a decrease in trabecular meshwork cells [60]. A different genetic mechanism underlying aqueous humor dynamics in different age categories may therefore explain the lack of association of IOP with rs11656696 and rs7555523 in these younger cohorts. In the TwinsUK cohort, 97.5% of the participants were women. Sex was not significantly related to IOP in the discovery cohorts. Moreover, the results from the TwinsUK strongly replicate the association of IOP with rs11656696, and are also supportive for the association with rs7555523. We therefore believe that the differences in sex have not substantially influenced our results. Finally, DCCT/EDIC comprised only participants with type I diabetes mellitus. DCCT/EDIC was the only cohort that showed an inconsistent effect for rs11656696. The reduced association in the joint analysis when compared to the discovery analysis (p-value increased from 9.8×10^−9^ to 1.4×10^−8^) was mainly driven by DCCT/EDIC. When DCCT/EDIC was not included, the association in the joint analysis became stronger than it was in the discovery (p-value decreased from 9.8×10^−9^ to p = 1.1×10^−9^). Although the association of type 1 diabetes with IOP is controversial, any changes in IOP may have different origins, which may explain the inconsistent replication results in this cohort.

In conclusion, this genome-wide association study in 8 independent Caucasian cohorts identified rs11656696 in *GAS7* at chromosome 17p13.1 and rs7555523 in *TMCO1* at chromosome 1q24.1 as common genetic variants associated with IOP. The variants were also marginally associated with glaucoma. *GAS7* and *TMCO1* are expressed in ocular cells and tissues implicated in glaucoma. Biochemical protein interactions with known glaucoma disease genes, as well as functional data support the involvement of these genes in aqueous humor dynamics and glaucomatous neuropathy.

## Materials and Methods

### Ethics statement

All participating studies adhered to the tenets of the Declaration of Helsinki and were approved by their Medical Ethics Committees. Written, informed consent was obtained from all participants.

### Outline of the study

For the gene discovery phase, we combined data of 11,972 participants derived from 4 large, independent population-based cohort studies in The Netherlands: the Rotterdam Study cohort I (RS-I), RS-II, RS-III, and the Erasmus Rucphen Family (ERF) Study. Replication of the findings was sought in 4 independent populations: the TwinsUK Adult Twin study, the Australian Twin Study, the Diabetes Control and Complications Trial / Epidemiology of Diabetes Interventions and Complications study (DCCT/EDIC) [61], and the Wellcome Trust Case-Control Consortium 2 / Blue Mountains Eye Study (WTCCC2/BMES). Clinical relevance of the identified loci was assessed by evaluating associations between the variants and glaucoma. To this end, we performed case-control analyses using 4 different glaucoma cohorts from The Netherlands and Germany. Finally, we examined the expression levels of the identified candidate genes in ocular tissues.

### Discovery studies

#### Participants

The RS-I is a prospective population-based cohort study of 7,983 residents 55 years of age and older living in Ommoord, a suburb of Rotterdam, The Netherlands [62]. Baseline ophthalmic examinations took place from 1991 to 1993, follow-up examinations from 1997 to 1999 and from 2002 to 2006. The RS-II is an independent cohort of another 3,011 new respondents in the same age range as RS-I [62]. Baseline examinations were performed from 2000 to 2002 and follow-up examinations from 2004 to 2005. The RS-III was based on the same protocol as RS-I and RS-II, and included 3,932 residents with a different age range, being 45 years and older. Baseline examinations took place from 2006 to 2009. Finally, ERF is a family-based cohort study in a genetically isolated population in the southwest of The Netherlands with over 3,000 participants 18 years of age and older [18], [63]. Examinations took place from 2002 to 2005.

#### IOP measurement

In all discovery cohorts, the IOP was measured with Goldmann applanation tonometry (Haag-Streit, Bern, Switzerland), which is the international standard for IOP assessment in ophthalmic research and clinical practice. A drop of fluorescein sodium was instilled in each eye. The tonometer was set at 10 mm Hg, and the prism was carefully applied to the corneal surface of the right eye. Without looking at the scale, the examiner rotated the dial until the inner margins of the two semicircles touched each other. The examiner then moved the slit lamp away from the eye and read the IOP. The tonometer was set at 10 mm Hg, and the measurement was repeated. If the two measurements differed, a third measurement was performed, and the median value was recorded. The procedure was repeated for the left eye [18], [64]. The IOP measurement was part of a comprehensive ophthalmic examination, including the assessment of visual acuity, refraction, keratometry, fundus photography, and imaging of the optic disc.

#### Genotyping

In the RS-I, RS-II and RS-III cohorts, DNA was genotyped with the Illumina Infinium II HumanHap550 chip v3.0 array. In the ERF study, DNA was genotyped on 4 different platforms (Illumina 6k, Illumina 318K, Illumina 370K and Affymetrix 250K), which were then merged. Genotype data were imputed by using HapMap CEU build 35 as the reference population, resulting in over 2.5 million SNPs. For details please see Text S1.

### Replication studies

SNPs showing strongest association in the discovery phase were carried forward and assessed for association with IOP in 2,235 participants from the TwinsUK Study, 1,807 from the Australian Twin Study, 1,304 from the DCCT/EDIC Study, and 2,136 from the WTCCC2/BMES Study. The TwinsUK , Australian Twin and WTCCC2/BMES were also population-based studies, and participants were ascertained regardless of their phenotypes or clinical status. The DCCT/EDIC study comprised only patients with type 1 diabetes included in a preventive trial. Descriptions of the study populations, clinical examinations, and genotyping methods of the replication cohorts are provided in Text S1 and Table S4.

#### Glaucoma case-control studies

SNPs showing the strongest associations in the discovery and replication phase were also evaluated in 4 series of glaucoma patients. The first series included 188 participants from RS-I in whom the technician measuring IOP was completely ignorant of the presence of glaucoma. Controls were healthy participants of RS-I. The second case-control study was an independent series of 104 glaucoma cases from an isolated population (Genetic Research in an Isolated Population [GRIP] study), with the ERF population as a control group. The third study included 152 cases and 141 controls recruited from all over The Netherlands as part of the Amsterdam Glaucoma Study (AGS). The last case-control study comprised a series of 988 glaucoma cases and 378 controls ascertained in Erlangen and Tübingen, Germany. Details of the clinical evaluation and glaucoma diagnosis in these studies are described in Text S1 and Table S5.

### Statistical analyses

#### Discovery analysis

Analyses were performed for the mean IOP of both eyes or for one eye if data on the other eye were missing. In the gene discovery analyses, IOP levels were imputed for those who received IOP lowering medication or had a history of IOP lowering surgery, because the initial IOP levels were unknown. Based on a reported average of a 30% IOP reduction caused by IOP lowering medication, estimated in a meta-analysis, IOP values of those receiving this medication were divided by 0.7 to estimate pre-treatment IOP [59]. In participants with a history of IOP lowering surgery, pre-treatment IOP was assumed to be at least 30 mmHg. The data were also analyzed after exclusion of any participants who received IOP lowering treatment or had a history of IOP lowering surgery. The results of these analyses have been presented in Text S1.

Associations between IOP and genome-wide loci were assessed with linear regression models under the assumption of an additive model for the effect of the risk allele. Analyses were adjusted for age and sex. In the ERF study, the analyses were also performed with additional adjustment for the time of the IOP measurement. Genomic inflation factors (λ) were calculated to evaluate any population stratification. Analyses were performed with the ProbABEL package from the ABEL set of programs (http://mga.bionet.nsc.ru/yurii/ABEL/) [65]. To adjust for familial relationships of participants in ERF, the score test for relatives was applied by using the genomic kinship matrix as implemented in the GenABEL package of R statistical software (http://cran.r-project.org) [65]–[67].

The results from the 4 cohorts were subjected to an inverse variance meta-analysis. Genomic control was used to correct the standard errors of the effect estimates before pooling [68]. The genome-wide threshold for statistical significance was set at a p-value of 5×10^−8^ to adjust for multiple testing [69]. Meta-analyses were performed with METAL software (http://www.sph.umich.edu/csg/abecasis/metal/index.html).

Results of the discovery meta-analysis were also used to explore regions in the immediate vicinity of the known glaucoma genes (*MYOC*, *OPTN*, *WDR36*) as well as the regions which had approached genome-wide significance in previous GWASs of glaucoma and previous linkage studies of IOP [9], [10], [13], [20], [24].

#### Replication analysis

Loci which were suggestive (p<1×10^−5^) of association with IOP in the discovery meta-analysis were taken forward to the replication phase. If two or more significantly associated SNPs within a locus were in linkage disequilibrium (LD), only the SNP with the best probability of association (lowest p-value) was selected. Linear regression analyses adjusted for age and sex were performed under the assumption of an additive effect of the risk allele. The results from the discovery and replication cohorts were combined by using an inverse variance meta-analysis (METAL software).

#### Glaucoma case-control analysis

SNPs that were genome-wide significantly associated with IOP in the meta-analysis of the discovery and replication cohorts were assessed in the 4 glaucoma case-control studies. Logistic regression analyses adjusted for age and sex were performed (SPSS version 15.0 for Windows; SPSS, Chicago, IL) and a pooled effect estimate was calculated (Rmeta software [http://cran.r-project.org/web/packages/rmeta/index.html]). Considering the difference in mean age between the cases and controls of the GRIP and ERF studies, the analyses were repeated after excluding any control subjects younger than 51 years of age from the ERF study. As this did not substantially change the odds ratios (from 0.99 [95%CI = 0.70–1.41] to 0.99 [95%CI = 0.70–1.42] for rs11656696, and from 1.78 [95%CI = 1.21–2.62] to 1.72 [95%CI = 1.15–2.57] for rs7555523), we only report the results of the initial analyses including all participants of the ERF study.

#### Protein pathway analyses

Protein pathway analysis was conducted in Ingenuity Knowledge Base (Ingenuity Systems, www.ingenuity.com). We looked for functional links between *GAS7* (*MLL* in rodents) and *TMCO1* and molecules known to play a role in glaucoma.

### Expression studies

Two independent expression studies were performed. In the first, retinal expression data were obtained essentially as described by Booij and colleagues [70]. Human healthy donor eyes (n = 4) were collected in collaboration with the Dutch Cornea Bank and snap frozen. History of the donor eyes revealed no glaucoma or other eye diseases. Cryosections (20 µm) of the CB were cut and mounted on PEN membrane slides (Carl Zeiss MicroImaging). With the use of laser dissection microscopy, the CB epithelium was cut out. RNA isolation (RNeasy Micro Kit, Qiagen) and amplification (Amino Allyl MessageAmp II aRNA Amplification, Ambion Applied Biosystems) were conducted according to the manufacturers' protocols. After labelling of experimental aRNA with Cy5 and reference aRNA (composed of RPE and choroid) with Cy3, we performed hybridization on catalogue human 4×44k microarrays (Agilent Technologies). Mean expression intensity data were normalized with R software (R Development Core Team, 2009). The mean expression data were further subdivided based on percentiles in Windows Excel. We used the 90^th^, 50^th^ and 10^th^ percentile of the mean expression intensity to categorize our data into groups with high (>90^th^), moderate (50^th^–90^th^), low (10^th^–50^th^) and very low (<10^th^) expression.

In the second expression study, ocular tissues were obtained for quantitative real-time PCR from four donor eyes (age: 81.2±4.5 years, 2 female, 2 male) without any known ocular disease. These eyes were obtained at autopsy and were processed within 8 hours after death. Informed consent to tissue donation was obtained from the donors or their relatives, and the protocol of the study was approved by the local Ethics Committee and adhered to the tenets of the Declaration of Helsinki for experiments involving human tissue. Total RNA was extracted from various ocular tissues by using the RNeasy kit (Quiagen, Hilden, Germany) including an on-column DNase I digestion step. First strand cDNA synthesis was performed by using 0.1 µg of total RNA, 200 U Superscript II reverse transcriptase (Invitrogen; Karlsruhe, Germany), and 500 ng oligo dT primers (Roche Diagnostics; Mannheim, Germany) in a 20 µl reaction volume. Quantitative real-time PCR was performed by means of the MyIQ thermal cycler and software (Biorad, Munich, Germany). PCR reactions (25 µl) contained 2 µl of first-strand cDNA, 0.4 µM each of upstream- and downstream-primer, 3.0 mM MgCl_2_, and 1× SsoFast EvaGreen Supermix (Biorad). All samples were analyzed in duplicates by means of a program with an initial denaturation step of 95°C for 3 minutes and 40 cycles of 95°C for 5 seconds, and 64°C (GAS7 and TMCO1) or 62°C (GAPDH) for 15 seconds. Gene-specific primers (Eurofins, Anzing, Germany) were designed to anneal with sequences located in different exons by means of Primer 3 software (http://fokker.wi.mit.edu/primer3/input.htm) and are summarized in Table S6. For quantification, serially diluted standard curves were run in parallel, and amplification specificity was checked using melt curve analysis. For normalization of gene expression levels, mRNA ratios relative to the house-keeping gene GAPDH were calculated.

## Supporting Information

Figure S1 QQ-plots for the observed versus expected p-values for the individual discovery cohorts and the discovery meta-analysis.
(DOC)

Figure S2 Regional association plots of loci associated with IOP (5×10^−8^<p-value<1×10^−5^) in meta-analysis.
(DOC)

Figure S3 Regional association plots of *MYOC*, *OPTN*, and *WDR36* regions in meta-analysis.
(DOC)

Table S1 Loci associated with IOP with p-values<10^−5^ after meta-analyses: results of individual cohorts. SNP = single nucleotide polymorphism; Chrom = Chromosome; MAF = minor allele frequency; SE = standard error; RS = Rotterdam Study; ERF = Erasmus Rucphen Family study.
(DOC)

Table S2 All SNPs associated with IOP with p-values<10^−5^ after meta-analyses. MA(F) = minor allele (frequency).
(DOC)

Table S3 Association results for SNPs identified in previous association studies. SNP = single nucleotide polymorphism; Chrom = Chromosome; SE = standard error; RS = Rotterdam Study; ERF = Erasmus Rucphen Family study.
(DOC)

Table S4 Characteristics of the replication cohorts. * not measured. **available for subset of 843 TwinsUK participants only, mean age 56 years. IOP = intraocular pressure; SD = standard deviation; DCCT/EDIC = Diabetes Control and Complications Trial / Epidemiology of Diabetes Interventions and Complications study; WTCCC/BMES = Wellcome Trust Case-Control Consortium / Blue Mountains Eye Study
(DOC)

Table S5 Characteristics of the glaucoma case-control studies. * not measured. IOP = intraocular pressure; SD = standard deviation; RS = Rotterdam Study; GRIP = Genetic Research in Isolated Populations; AGS = Amsterdam Glaucoma Study.
(DOC)

Table S6 PCR-primers used for the expression study. T_an_, annealing temperature.
(DOC)

Text S1 Additional Methodology: Detailed information on genotyping and imputation methods of discovery cohorts, description of methodology replication cohorts, and description of methodology case-control studies. Additional Results: Results of the discovery analyses after exclusion of any participants who received IOP lowering treatment or who had received this treatment in the past.
(DOC)
