# Supplementary material for: Common Genetic Determinants of Intraocular Pressure and Primary Open-Angle Glaucoma
Source: PLoS Genet. 2012 May 3;8(5):e1002611. doi: 10.1371/journal.pgen.1002611 (PMC3342933; doi:10.1371/journal.pgen.1002611)
Supplement: Figure S1 — QQ-plots for the observed versus expected p-values for the individual discovery cohorts and the discovery meta-analysis. (DOC) [file pgen.1002611.s001.doc]

**Figure S1.** QQ-plots for the observed versus expected p-values for the individual discovery cohorts and the discovery meta-analysis

| RS-I  **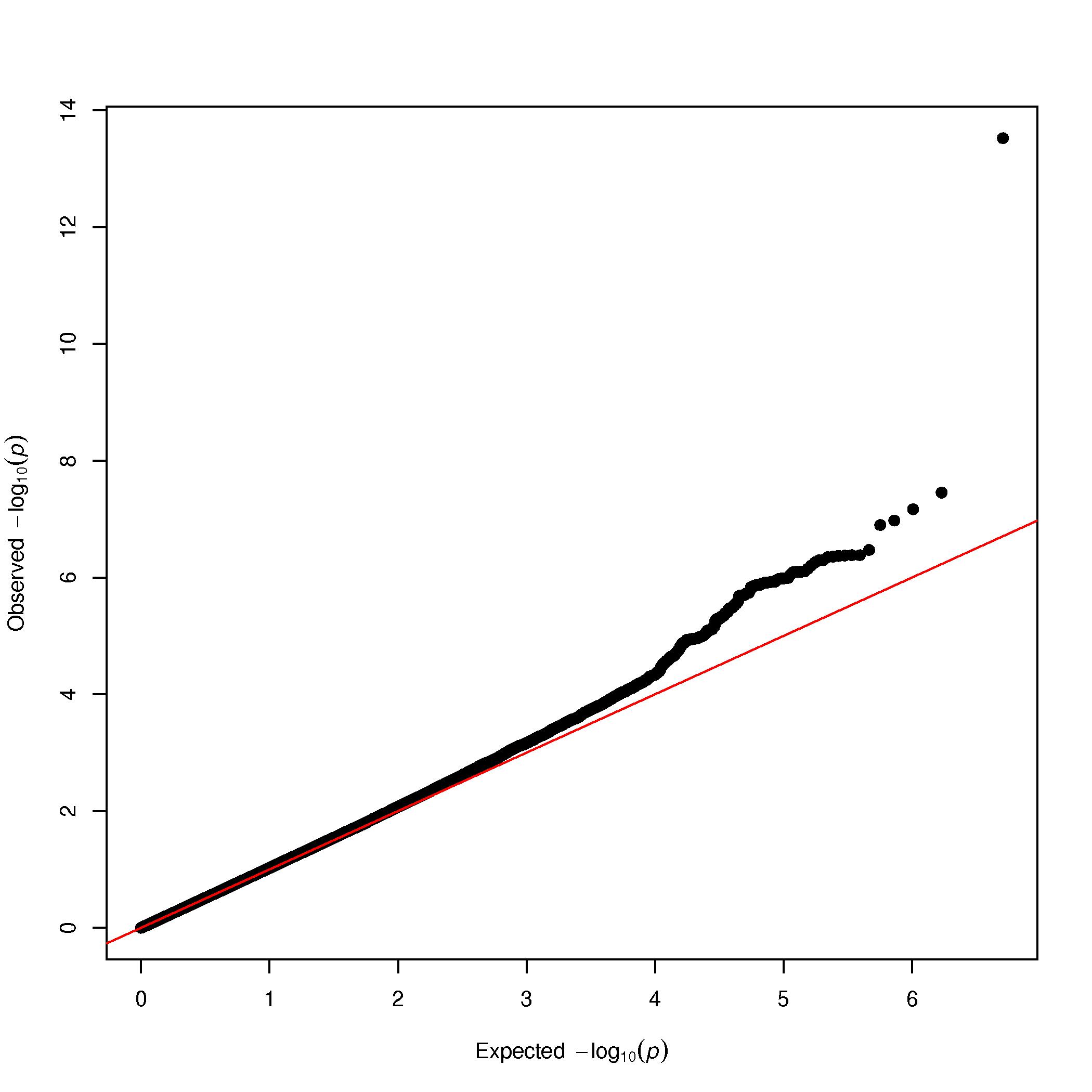** | RS-II  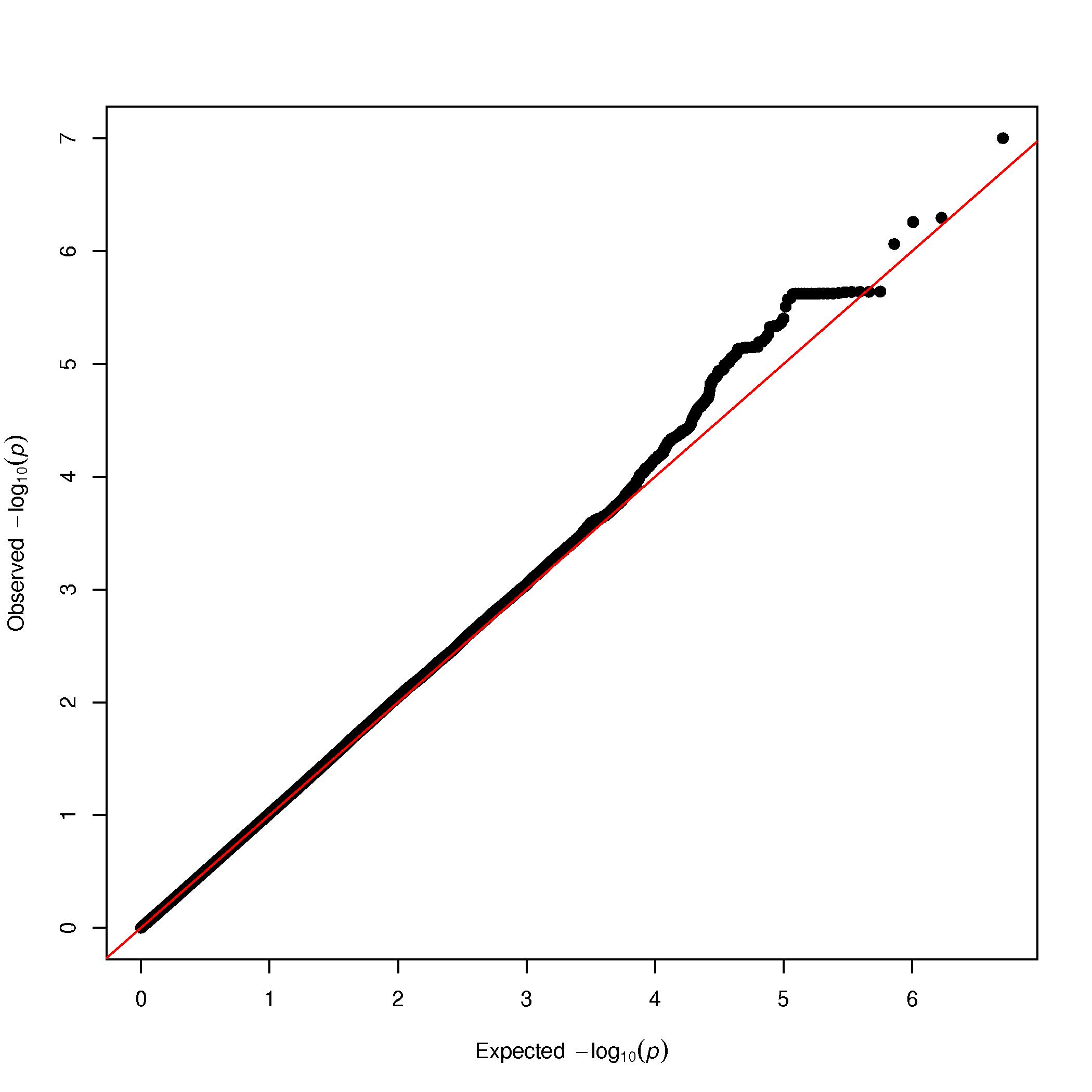 |
| --- | --- |
| RS-III  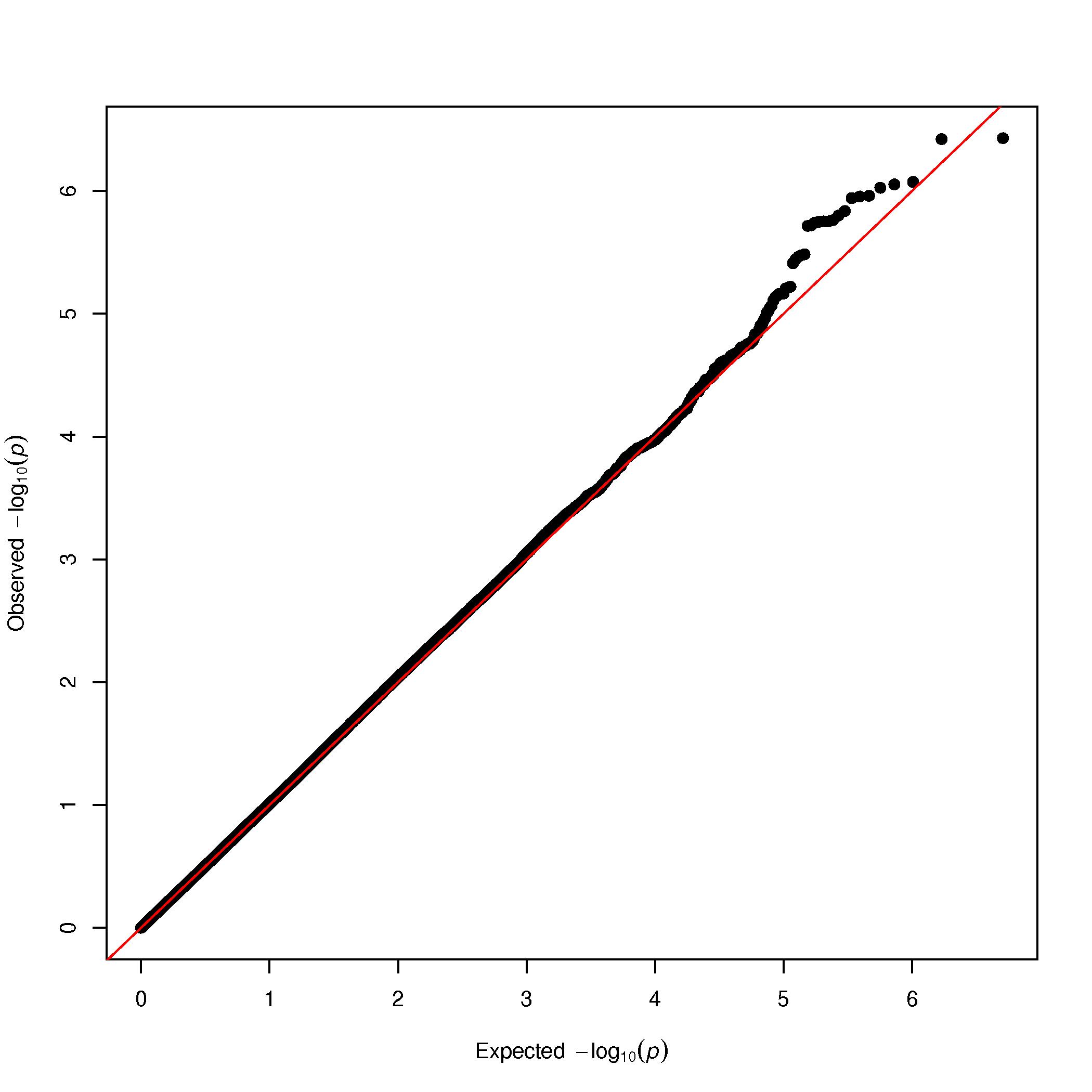 | ERF  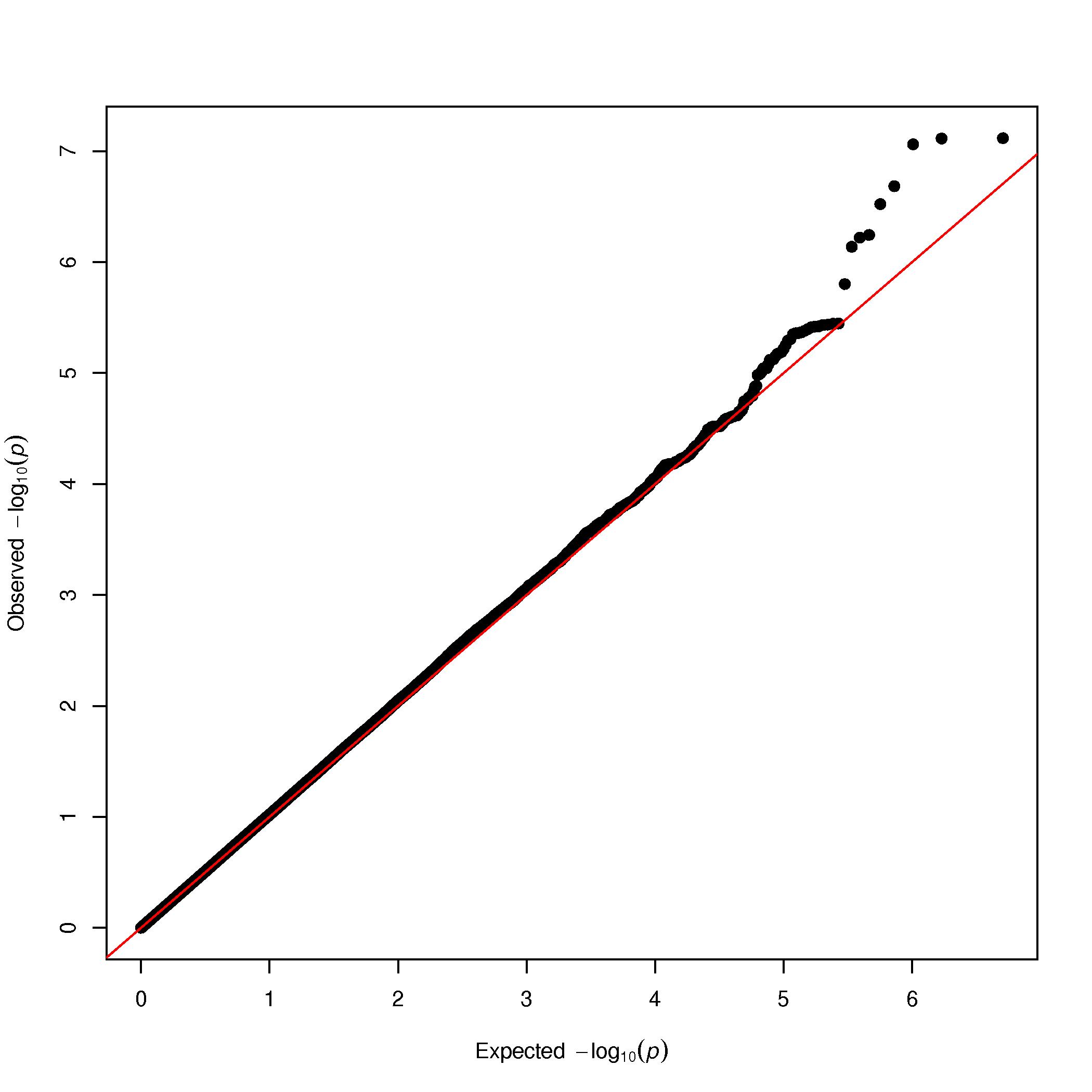 |
| Discovery meta-analysis  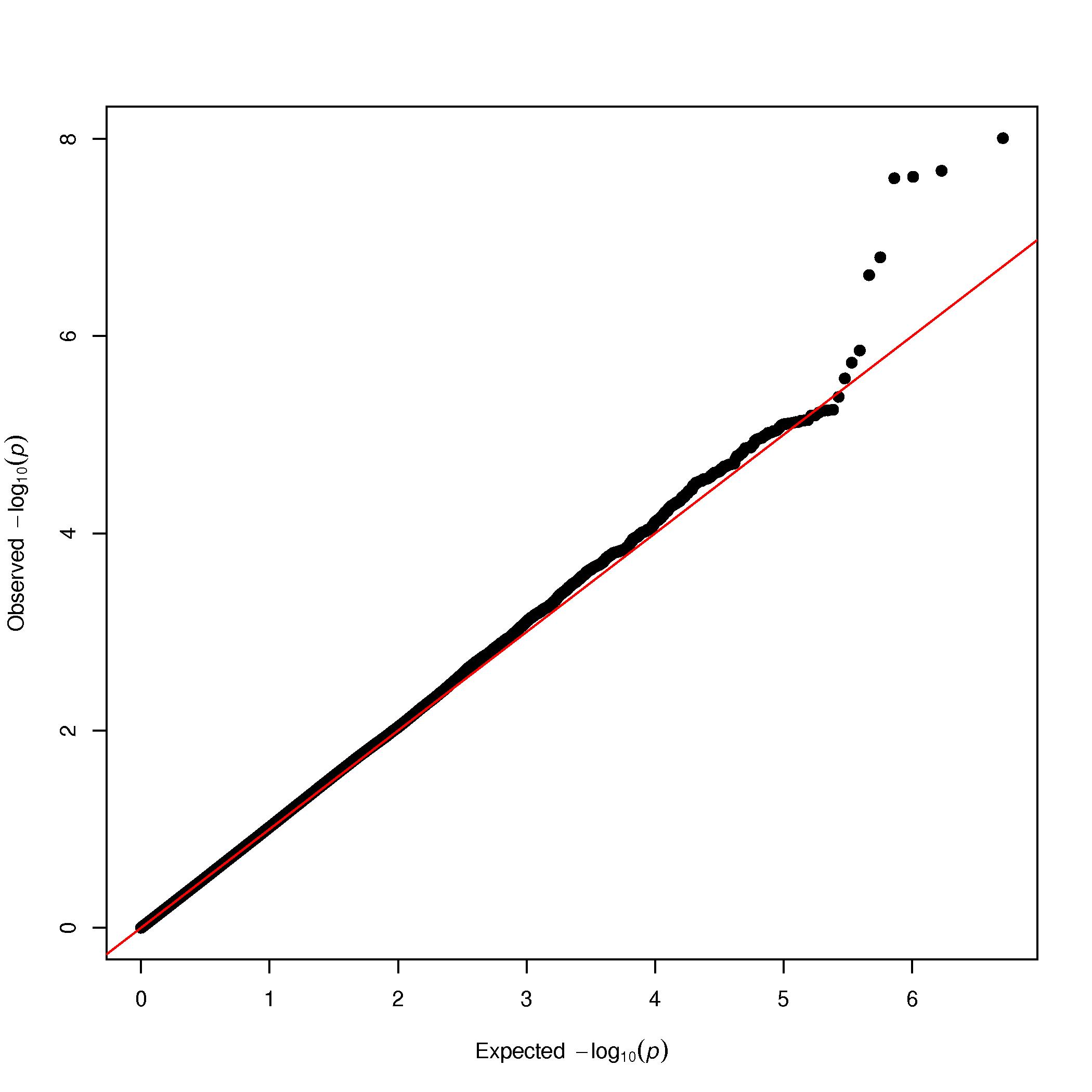 |  |
