# Supplementary material for: Common Genetic Determinants of Intraocular Pressure and Primary Open-Angle Glaucoma
Source: PLoS Genet. 2012 May 3;8(5):e1002611. doi: 10.1371/journal.pgen.1002611 (PMC3342933; doi:10.1371/journal.pgen.1002611)
Supplement: Figure S2 — Regional association plots of loci associated with IOP (5×10^−8^<p-value<1×10^−5^) in meta-analysis. (DOC) [file pgen.1002611.s002.doc]

**Figure S2. Regional association plots of loci associated with IOP (5x10^-8^ < p-value < 1x10^-5^) in meta-analysis**

|  |  |
| --- | --- |
|  |  |
|  |  |
