# Supplementary material for: Common Genetic Determinants of Intraocular Pressure and Primary Open-Angle Glaucoma
Source: PLoS Genet. 2012 May 3;8(5):e1002611. doi: 10.1371/journal.pgen.1002611 (PMC3342933; doi:10.1371/journal.pgen.1002611)
Supplement: Figure S3 — Regional association plots of MYOC, OPTN, and WDR36 regions in meta-analysis. (DOC) [file pgen.1002611.s003.doc]

**Figure S3. Regional association plots of *MYOC*, *OPTN*, and *WDR36* regions in meta-analysis**

|  |
| --- |
|  |
|  |
