# Supplementary material for: Common Genetic Determinants of Intraocular Pressure and Primary Open-Angle Glaucoma
Source: PLoS Genet. 2012 May 3;8(5):e1002611. doi: 10.1371/journal.pgen.1002611 (PMC3342933; doi:10.1371/journal.pgen.1002611)
Supplement: Table S1 — Loci associated with IOP with p-values<10−5 after meta-analyses: results of individual cohorts. SNP = single nucleotide polymorphism; Chrom = Chromosome; MAF = minor allele frequency; SE = standard error; RS = Rotterdam Study; ERF = Erasmus Rucphen Family study. (DOC) [file pgen.1002611.s004.doc]

**Table S1. Loci associated with IOP with p-values <10^-5^ after meta-analyses: results of individual cohorts**

| SNP | Chrom | RS-I |  |  |  | RS-II |  |  |  | RS-III |  |  |  | ERF |  |  |  |
| --- | --- | --- | --- | --- | --- | --- | --- | --- | --- | --- | --- | --- | --- | --- | --- | --- | --- |
|  |  | MAF | Beta | SE | P-value | MAF | Beta | SE | P-value | MAF | Beta | SE | P-value | MAF | Beta | SE | P-value |
| rs11656696 | 17p13.1 | 0.43 | -0.351 | 0.064 | 3.4x10^-8^ | 0.43 | -0.107 | 0.107 | 3.1x10^-1^ | 0.43 | -0.241 | 0.101 | 1.7x10^-2^ | 0.39 | -0.156 | 0.120 | 1.9x10^-1^ |
| rs7894966 | 10q23.2 | 0.04 | 0.909 | 0.195 | 3.0x10^-6^ | 0.04 | 0.226 | 0.308 | 4.6x10^-1^ | 0.04 | 0.561 | 0.267 | 3.6x10^-2^ | 0.04 | 0.697 | 0.300 | 2.0x10^-2^ |
| rs216146 | 5q32 | 0.39 | 0.238 | 0.067 | 3.7x10^-4^ | 0.40 | 0.115 | 0.110 | 3.0x10^-1^ | 0.38 | 0.247 | 0.098 | 1.2x10^-2^ | 0.39 | 0.252 | 0.113 | 2.6x10^-2^ |
| rs2117760 | 3p13 | 0.32 | 0.242 | 0.070 | 5.5x10^-4^ | 0.32 | 0.336 | 0.114 | 3.2x10^-3^ | 0.32 | 0.130 | 0.104 | 2.1x10^-1^ | 0.33 | 0.150 | 0.112 | 1.8x10^-1^ |
| rs7555523 | 1q24.1 | 0.12 | 0.331 | 0.097 | 6.2x10^-4^ | 0.12 | 0.515 | 0.162 | 1.5x10^-3^ | 0.13 | 0.223 | 0.142 | 1.2x10^-1^ | 0.10 | 0.100 | 0.166 | 5.5x10^-1^ |
| rs1826598 | 16q23.1 | 0.11 | 0.517 | 0.103 | 5.4x10^-7^ | 0.10 | 0.185 | 0.171 | 2.8x10^-1^ | 0.10 | 0.127 | 0.157 | 4.2x10^-1^ | 0.10 | 0.164 | 0.177 | 3.5x10^-1^ |
| rs9841621 | 3p24.3 | 0.01 | -1.079 | 0.277 | 1.0x10^-4^ | 0.01 | -0.434 | 0.466 | 3.5x10^-1^ | 0.01 | -0.777 | 0.422 | 6.5x10^-2^ | 0.02 | -0.620 | 0.368 | 9.2x10^-2^ |
