# Supplementary material for: Common Genetic Determinants of Intraocular Pressure and Primary Open-Angle Glaucoma
Source: PLoS Genet. 2012 May 3;8(5):e1002611. doi: 10.1371/journal.pgen.1002611 (PMC3342933; doi:10.1371/journal.pgen.1002611)
Supplement: Table S2 — All SNPs associated with IOP with p-values<10−5 after meta-analyses. MA(F) = minor allele (frequency). (DOC) [file pgen.1002611.s005.doc]

**Table S2. All SNPs associated with IOP with p-values<10^-5^ after meta-analyses**

| SNP | Chromosome | Position | MA | MAF | Beta | SE | Pvalue |
| --- | --- | --- | --- | --- | --- | --- | --- |
| rs11656696 | 17 | 9974404 | A | 0.43 | -0.26 | 0.05 | 9.8E-09 |
| rs9889528 | 17 | 9976062 | T | 0.41 | -0.26 | 0.05 | 2.1E-08 |
| rs9913911 | 17 | 9971908 | G | 0.37 | -0.25 | 0.05 | 2.4E-08 |
| rs12150284 | 17 | 9971815 | T | 0.37 | -0.25 | 0.05 | 2.5E-08 |
| rs7894966 | 10 | 88608604 | G | 0.04 | 0.67 | 0.13 | 1.6E-07 |
| rs6483184 | 11 | 92028230 | C | 0.01 | 1.70 | 0.33 | 2.4E-07 |
| rs216146 | 5 | 149426114 | T | 0.39 | 0.22 | 0.05 | 1.4E-06 |
| rs42639 | 7 | 89808659 | A | 0.00 | 6.48 | 1.36 | 1.9E-06 |
| rs216150 | 5 | 149427821 | A | 0.35 | 0.22 | 0.05 | 2.7E-06 |
| rs2117760 | 3 | 70933151 | A | 0.32 | 0.22 | 0.05 | 4.1E-06 |
| rs17107107 | 10 | 88595222 | T | 0.02 | 0.74 | 0.16 | 5.6E-06 |
| rs6745633 | 2 | 206427798 | C | 0.00 | 3.18 | 0.70 | 5.7E-06 |
| rs7555523 | 1 | 163985603 | C | 0.12 | 0.30 | 0.07 | 5.7E-06 |
| rs1826598 | 16 | 76130456 | A | 0.11 | 0.32 | 0.07 | 6.0E-06 |
| rs6696454 | 1 | 163974547 | T | 0.12 | 0.30 | 0.07 | 6.4E-06 |
| rs4657476 | 1 | 163999285 | C | 0.12 | 0.30 | 0.07 | 6.4E-06 |
| rs11814254 | 10 | 88596918 | A | 0.02 | 0.73 | 0.16 | 7.1E-06 |
| rs4114847 | 10 | 88591590 | A | 0.02 | 0.74 | 0.16 | 7.2E-06 |
| rs12762647 | 10 | 88593125 | G | 0.02 | 0.73 | 0.16 | 7.2E-06 |
| rs2814471 | 1 | 164006222 | C | 0.12 | 0.30 | 0.07 | 7.4E-06 |
| rs6660601 | 1 | 163962479 | C | 0.12 | 0.30 | 0.07 | 7.5E-06 |
| rs2790052 | 1 | 164005087 | G | 0.12 | 0.30 | 0.07 | 7.6E-06 |
| rs2251768 | 1 | 164004935 | A | 0.12 | 0.30 | 0.07 | 7.7E-06 |
| rs2790053 | 1 | 164004328 | C | 0.12 | 0.30 | 0.07 | 7.7E-06 |
| rs7524755 | 1 | 163961521 | T | 0.12 | 0.30 | 0.07 | 7.8E-06 |
| rs7518099 | 1 | 164003504 | C | 0.12 | 0.30 | 0.07 | 7.8E-06 |
| rs12243345 | 10 | 88603090 | G | 0.02 | 0.72 | 0.16 | 8.0E-06 |
| rs12778494 | 10 | 88606511 | C | 0.02 | 0.72 | 0.16 | 8.5E-06 |
| rs9841621 | 3 | 18384081 | G | 0.01 | -0.81 | 0.18 | 8.9E-06 |
| rs9879573 | 3 | 18384173 | T | 0.01 | -0.81 | 0.18 | 9.2E-06 |
| rs9879688 | 3 | 18384071 | A | 0.01 | -0.81 | 0.18 | 9.2E-06 |
| rs9837588 | 3 | 18383934 | G | 0.01 | -0.81 | 0.18 | 9.4E-06 |
| rs4656461 | 1 | 163953829 | G | 0.13 | 0.29 | 0.07 | 9.6E-06 |
| rs9713190 | 3 | 18383100 | C | 0.01 | -0.81 | 0.18 | 9.6E-06 |
| rs12772861 | 10 | 88518342 | C | 0.02 | 0.74 | 0.17 | 1.0E-05 |
