# Supplementary material for: Common Genetic Determinants of Intraocular Pressure and Primary Open-Angle Glaucoma
Source: PLoS Genet. 2012 May 3;8(5):e1002611. doi: 10.1371/journal.pgen.1002611 (PMC3342933; doi:10.1371/journal.pgen.1002611)
Supplement: Table S3 — Association results for SNPs identified in previous association studies. SNP = single nucleotide polymorphism; Chrom = Chromosome; SE = standard error; RS = Rotterdam Study; ERF = Erasmus Rucphen Family study. (DOC) [file pgen.1002611.s006.doc]

**Table S3. Association results for SNPs identified in previous association studies**

| SNP [allele] | Chrom | Freq | Discovery meta-analysis | | | RS-I |  |  | RS-II |  |  | RS-III |  |  | ERF |  |  |  |
| --- | --- | --- | --- | --- | --- | --- | --- | --- | --- | --- | --- | --- | --- | --- | --- | --- | --- | --- |
|  |  |  | Beta | SE | P-value | Beta | SE | P-value | Beta | SE | P-value | Beta | SE | P-value | Beta | SE | P-value | |
| ***Nakano et al*** |  |  |  |  |  |  |  |  |  |  |  |  |  |  |  |  |  | |
| rs547984[C] | 1q43 | 0.55 | 0.03 | 0.04 | 4.9x10^-01^ | 0.03 | 0.06 | 6.8x10^-01^ | -0.17 | 0.11 | 9.8x10^-02^ | 0.06 | 0.10 | 5.1x10^-01^ | 0.21 | 0.11 | 4.8x10^-02^ | |
| rs540782[G] | 1q43 | 0.57 | 0.04 | 0.04 | 3.6x10^-01^ | 0.04 | 0.06 | 5.0x10^-01^ | -0.17 | 0.11 | 1.1x10^-01^ | 0.08 | 0.10 | 3.8x10^-01^ | 0.19 | 0.11 | 7.2x10^-02^ | |
| rs693421[G] | 1q43 | 0.57 | 0.04 | 0.04 | 3.5x10^-01^ | 0.04 | 0.06 | 4.9x10^-01^ | -0.17 | 0.11 | 1.2x10^-01^ | 0.09 | 0.10 | 3.6x10^-01^ | 0.19 | 0.11 | 7.3x10^-02^ | |
| rs2499601[T] | 1q43 | 0.46 | 0.06 | 0.04 | 1.9x10^-01^ | 0.08 | 0.06 | 2.3x10^-01^ | -0.13 | 0.10 | 2.0x10^-01^ | 0.17 | 0.09 | 7.5x10^-02^ | 0.06 | 0.11 | 5.5x10^-01^ | |
| rs7081455[G] | 10p12.31 | 0.45 | 0.12 | 0.04 | 4.6x10^-03^ | 0.16 | 0.06 | 1.4x10^-02^ | 0.02 | 0.11 | 8.4x10^-01^ | 0.15 | 0.10 | 1.3x10^-01^ | 0.11 | 0.11 | 2.9x10^-01^ | |
| rs7961953[G] | 12q21.31 | 0.88 | 0.01 | 0.07 | 8.5x10^-01^ | 0.07 | 0.10 | 4.7x10^-01^ | -0.25 | 0.16 | 1.2x10^-01^ | -0.06 | 0.15 | 6.9x10^-01^ | 0.18 | 0.15 | 2.2x10^-01^ | |
|  |  |  |  |  |  |  |  |  |  |  |  |  |  |  |  |  |  | |
| ***Jiao et al*** |  |  |  |  |  |  |  |  |  |  |  |  |  |  |  |  |  | |
| rs1533428[T] | 2p16 | 0.30 | 0.02 | 0.05 | 7.0x10^-01^ | 0.02 | 0.07 | 7.6x10^-01^ | 0.09 | 0.12 | 4.5x10^-01^ | 0.10 | 0.10 | 3.4x10^-01^ | -0.16 | 0.12 | 1.6x10^-01^ | |
| rs12994401[T] | 2p16 | 0.19 | -0.04 | 0.05 | 4.8x10^-01^ | 0.04 | 0.08 | 6.2x10^-01^ | -0.14 | 0.13 | 2.8x10^-01^ | -0.02 | 0.12 | 8.8x10^-01^ | -0.16 | 0.13 | 2.1x10^-01^ | |
|  |  |  |  |  |  |  |  |  |  |  |  |  |  |  |  |  |  | |
| ***Thorleifsson et al*** |  |  |  |  |  |  |  |  |  |  |  |  |  |  |  |  |  | |
| rs4236601[A] | 7q31 | 0.29 | 0.19 | 0.05 | 1.1x10^-04^ | 0.24 | 0.07 | 7.6x10^-04^ | 0.08 | 0.11 | 4.7x10^-01^ | 0.27 | 0.11 | 1.1x10^-02^ | 0.05 | 0.12 | 6.9x10^-01^ | |
| rs1052990[G] | 7q31 | 0.36 | 0.17 | 0.04 | 1.6x10^-04^ | 0.22 | 0.07 | 8.7x10^-04^ | 0.00 | 0.11 | 9.7x10^-01^ | 0.26 | 0.10 | 8.9x10^-03^ | 0.11 | 0.11 | 3.2x10^-01^ | |
|  |  |  |  |  |  |  |  |  |  |  |  |  |  |  |  |  |  | |
| ***Burdon et al*** |  |  |  |  |  |  |  |  |  |  |  |  |  |  |  |  |  | |
| rs4656461[G] | 1q24 | 0.13 | 0.29 | 0.07 | 9.6x10^-6^ | 0.32 | 0.10 | 8.0x10^-4^ | 0.51 | 0.16 | 1.6x10^-3^ | 0.21 | 0.14 | 1.3x10^-1^ | 0.09 | 0.17 | 5.9x10^-1^ | |
| Rs4977756[A] | 9p21 | 0.58 | 0.05 | 0.04 | 2.5x10^-1^ | 0.02 | 0.06 | 7.2x10^-1^ | 0.11 | 0.11 | 3.2x10^-1^ | -0.01 | 0.10 | 9.3x10^-1^ | 0.13 | 0.10 | 2.0x10^-1^ | |
