# Supplementary material for: Common Genetic Determinants of Intraocular Pressure and Primary Open-Angle Glaucoma
Source: PLoS Genet. 2012 May 3;8(5):e1002611. doi: 10.1371/journal.pgen.1002611 (PMC3342933; doi:10.1371/journal.pgen.1002611)
Supplement: Table S4 — Characteristics of the replication cohorts. * not measured. **available for subset of 843 TwinsUK participants only, mean age 56 years. IOP = intraocular pressure; SD = standard deviation; DCCT/EDIC = Diabetes Control and Complications Trial / Epidemiology of Diabetes Interventions and Complications study; WTCCC/BMES = Wellcome Trust Case-Control Consortium / Blue Mountains Eye Study (DOC) [file pgen.1002611.s007.doc]

**Table S4. Characteristics of the replication cohorts**

| Characteristic | TwinsUK | Australian Twins | DCCT/EDIC | WTCCC2/BMES |
| --- | --- | --- | --- | --- |
| Participants with valid data (N) | 2,235 | 1,807 | 1,304 | 2,136 |
| Age (y), mean ± SD (range) | 56.8 ±11.7 (16– 83) | 22.2 ± 12.7 (5 – 90) | 26.8 ± 7.1 (13 - 39) | 62.8 ± 8.2 (49 – 91) |
| Male gender (%) | 2.5 | 44 | 53 | 43 |
| IOP (mmHg), mean ± SD (range) | 15.6 ± 3.1 (6.5 – 30) | 15.8 ± 3.0 (6 – 30) | 15.7 ± 2.7 (9 - 22) | 15.5 ± 2.8 (8 - 27) |
| IOP ≥ 22 mmHg (%) | 3.3 | 1.3 | 1.0 | 1.9 |
| Participants with IOP lowering treatment (%) | 1 | 0 | 0 | 0 |
| Disc area (mm^2^), mean ± SD (range) | 2.6 ± 0.7 (0.7-7.0)** | 2.1 ± 0.4 (1.1-3.6) | * | * |
| Vertical cup-disc ratio, mean ± SD (range) | 0.32 ± 0.10 (0.07-0.70)** | 0.45 ± 0.13 (0.09-0.88) | * | 0.41 ± 0.14 (0.07-0.95) |
