# Supplementary material for: Common Genetic Determinants of Intraocular Pressure and Primary Open-Angle Glaucoma
Source: PLoS Genet. 2012 May 3;8(5):e1002611. doi: 10.1371/journal.pgen.1002611 (PMC3342933; doi:10.1371/journal.pgen.1002611)
Supplement: Table S5 — Characteristics of the glaucoma case-control studies. * not measured. IOP = intraocular pressure; SD = standard deviation; RS = Rotterdam Study; GRIP = Genetic Research in Isolated Populations; AGS = Amsterdam Glaucoma Study. (DOC) [file pgen.1002611.s008.doc]

**Table S5**. Characteristics of the glaucoma case-control studies

|  | RS-I |  | GRIP |  | AGS |  | Erlangen and Tübingen | |
| --- | --- | --- | --- | --- | --- | --- | --- | --- |
|  | Cases | Controls | Cases | Controls | Cases | Controls | Cases | Controls |
| Number of participants | 188 | 5,548 | 104 | 2,035 | 152 | 141 | 988 | 378 |
| Age (y), mean ± SD  (range) | 75.5 ± 7.4  (56 - 94) | 74.5 ± 7.8  (55 - 105) | 73.3 ± 9.2  (51 – 91) | 48.8 ± 14.4  (18 – 86) | 73.1 ± 11.1  (27 – 98) | 72.2 ± 8.2  (55 – 92) | 66.5 ± 14.1  (12 – 104) | 73.9 ± 6.4  (34 – 97) |
| Male gender (%) | 54.8 | 40.7 | 47.1 | 43.3 | 54.4 | 43.7 | 39.0 | 40.2 |
| IOP (mmHg), mean ± SD  (range) | 18.2 ± 6.2  (6 – 55) | 15.2 ± 3.5  (5 – 59) | 25.9 ± 8.6  (12 – 62) | 15.3 ± 3.1  (6 – 33) | 27.0 ± 8.3  (13 – 54) | 19.6 ± 7.7  (13 – 48) | 27.8 ± 9.3  (11 – 65) | <21.0 |
| IOP ≥ 22 mm Hg (%) | 20.7 | 3.3 | 60.6 | 1.2 | 73.3 | * | 50.8 | 0 |
| Participants with IOP lowering treatment (%) | 19.7 | 1.7 | 100.0 | 0.9 | 100.0 | 0.0 | 99.0 | 0.0 |
