# Supplementary material for: Common Genetic Determinants of Intraocular Pressure and Primary Open-Angle Glaucoma
Source: PLoS Genet. 2012 May 3;8(5):e1002611. doi: 10.1371/journal.pgen.1002611 (PMC3342933; doi:10.1371/journal.pgen.1002611)
Supplement: Table S6 — PCR-primers used for the expression study. Tan, annealing temperature. (DOC) [file pgen.1002611.s009.doc]

**Table S6. PCR-primers used for the expression study**

Gene Accession No. Product T_an_  MgCl_2_ Sequence (5‘ - 3‘)

GAS7 NM_201432 185 bp 64°C 3.0 mM TGGAGATCAAGCTGAGCAACAAG

CATCTCTACCCTCTCCACCTCCA

TMCO1 NM_019026 205 bp 64°C 3.0 mM GAAGGTGCGAGATGAGCACTATG

TGTTGTCGACCAGCTGACTCTGT

GAPDH NM_002046 127 bp 62°C 3.0 mM TCAACAGCGACACCCACTCCTC

ATGAGGTCCACCACCCTGTTGC
