# Supplementary material for: Common Genetic Determinants of Intraocular Pressure and Primary Open-Angle Glaucoma
Source: PLoS Genet. 2012 May 3;8(5):e1002611. doi: 10.1371/journal.pgen.1002611 (PMC3342933; doi:10.1371/journal.pgen.1002611)
Supplement: Text S1 — Additional Methodology: Detailed information on genotyping and imputation methods of discovery cohorts, description of methodology replication cohorts, and description of methodology case-control studies. Additional Results: Results of the discovery analyses after exclusion of any participants who received IOP lowering treatment or who had received this treatment in the past. (DOC) [file pgen.1002611.s010.doc]

**Supporting Information for**

**Common Genetic Determinants of Intraocular Pressure and Primary Open-Angle Glaucoma**

Leonieke M.E. van Koolwijk,^1,2^* Wishal D. Ramdas,^2,3^* M. Kamran Ikram,^2,3,4^ Nomdo M. Jansonius,^2,5^ Francesca Pasutto,^6^ Pirro G. Hysi,^7^ Stuart Macgregor,^8^ Sarah F.Janssen,^9^ Alex W. Hewitt,^10^ Ananth C Viswanathan,^11^ Jacoline B. ten Brink,^9^ S. Mohsen Hosseini,^12^ Najaf Amin,^2^ Dominiek D.G. Despriet,^2,3^ Jacqueline J.M. Willemse-Assink,^13^ Rogier Kramer,^14^ Fernando Rivadeneira,^2,15^ Maksim Struchalin,^2^ Yurii S. Aulchenko,^2^ Nicole Weisschuh,^16^ Matthias Zenkel,^17^ Christian Y. Mardin,^17^ Eugen Gramer,^18^ Ulrich Welge-Lüssen,^19^ Grant W. Montgomery,^8^ Francis Carbonaro,^7^ Terri L. Young,^20^ the DCCT/EDIC research group, Céline Bellenguez,^21^ Peter McGuffin,^22^ Paul J. Foster,^11^ Fotis Topouzis,^23^ Paul Mitchell,^24^ Jie Jin Wang,^24^ Tien Y. Wong,^10,25,26^ Monika A. Czudowska,^2,3^ Albert Hofman,^2^ Andre G. Uitterlinden,^2,15^ Roger C.W. Wolfs,^2,3^ Paulus T.V.M. de Jong,^9,27^ Ben A. Oostra,^28^ Andrew D. Paterson,^12,29^ Wellcome Trust Case Control Consortium 2,^30^ David A. Mackey,^31^ Arthur A.B. Bergen,^9,27,32^ André Reis,^6^ Christopher J. Hammond,^7^ Johannes R. Vingerling,^2,3^ Hans G. Lemij,^1^ Caroline C.W. Klaver,^2,3^ Cornelia M. van Duijn^2^

* These authors contributed equally

Authors affiliations:

^1^Glaucoma Service, The Rotterdam Eye Hospital, Rotterdam; Departments of ^2^Epidemiology, ^3^Ophthalmology, and ^4^Neurology, The Erasmus University Medical Center, Rotterdam; ^5^Department of Ophthalmology, University Medical Center Groningen, University of Groningen, Groningen – all in The Netherlands; ^6^Institute of Human Genetics, University Erlangen-Nuremberg, Erlangen, Germany; ^7^Department of Twin Research and Genetic Epidemiology, King’s College London, London, United Kingdom; ^8^Queensland Institute of Medical Research, Brisbane, Australia; ^9^Department of Molecular Ophthalmogenetics, The Netherlands Institute for Neuroscience (NIN), Royal Netherlands Academy of Arts and Sciences (KNAW), Amsterdam, The Netherlands; ^10^Centre for Eye Research Australia, University of Melbourne, Royal Victorian Eye and Ear Hospital, Melbourne, Australia; ^11^NIHR Biomedical Research Centre for Ophthalmology, Moorfields Eye Hospital NHS Foundation Trust and UCL Institute of Ophthalmology, London, United Kingdom; ^12^Program in Genetics and Genome Biology, Hospital for Sick Children, Toronto, Canada; ^13^Department of Ophthalmology, Amphia Hospital, Breda, The Netherlands; ^14^Department of Ophthalmology, Franciscus Hospital, Roosendaal, The Netherlands; ^15^Department of Internal Medicine, The Erasmus University Medical Center, Rotterdam, The Netherlands;^16^Molecular Genetics Laboratory, University Eye Hospital, Tübingen, Germany; ^17^Department of Ophthalmology, University of Erlangen-Nuremberg, Erlangen, Germany; ^18^University Eye Hospital, Würzburg, Germany; ^19^Department of Ophthalmology, Friedrich-Alexander University, Erlangen, Germany; ^20^Center for Human Genetics, Duke University, Durham, North Carolina, USA; ^21^Wellcome Trust Centre for Human Genetics, Oxford, United Kingdom; ^22^MRC Social Genetic and Developmental Psychiatry Research Centre, Institute of Psychiatry, King's College, London, United Kingdom; ^23^Department of Ophthalmology, School of Medicine, Aristotle University of Thessaloniki, AHEPA Hospital, Thessaloniki, Greece; ^24^Centre for Vision Research, University of Sydney, Sydney, Australia; ^25^Singapore National Eye Centre & Singapore Eye Research Institute, Singapore; ^26^Yong Loo Lin School of Medicine, National University of Singapore, Singapore; ^27^Department of Ophthalmology, Academic Medical Center, Amsterdam, The Netherlands; ^28^Department of Clinical Genetics, The Erasmus University Medical Center, Rotterdam, The Netherlands; ^29^Dalla Lana School of Public Health, University of Toronto, Toronto, Canada; ^30^Full membership of WTCCC2 is listed at the end of this article; ^31^Lions Eye Institute, University of Western Australia, Centre for Ophthalmology and Visual Science, Perth, Australia

^32^Department of Clinical Genetics, Academic Medical Center, Amsterdam, the Netherlands.

**Additional Methodology**

**Genotyping and imputation methods discovery cohorts**

In the three cohorts from the Rotterdam Study, DNA was genotyped with the Illumina Infinium II HumanHap550 chip v3.0 array. In the ERF study, DNA was genotyped on four different platforms (Illumina 6k, Illumina 318K, Illumina 370K and Affymetrix 250K), which were then merged. Participants with low call rate (<97.5%), excess heterozygosity (>0.336), or mismatch between reported and genetically determined sex were excluded from the analysis. For each study, single-nucleotide polymorphisms (SNPs) were filtered to satisfy a call rate >98% and a Hardy-Weinberg equilibrium test p-value >1x10^-5^. Genotype data were imputed by using HapMap CEU build 35 as the reference population, resulting in over 2.5 million SNPs (Markov Chain Haplotyping (MaCH) package; <http://www.sph.umich.edu/csg/abecasis/MACH>). After quality control, a total of 5,974 participants from RS-I, 2,157 (RS-II), 2,082 (RS-III), and 2,385 (ERF) had valid genotype data.

**Replication Cohorts**

Descriptive characteristics of the replication cohorts are presented in Table S3.

*TwinsUK Adult Twin study*

Participants were recruited from the TwinsUK Adult Twin Registry, based at St. Thomas’ Hospital, London. They were twin volunteers from the general population, and were part of a twin study on glaucoma heritability. Intraocular pressure (IOP) was measured by using the Ocular Response Analyser (ORA-Reichert®, Buffalo, NY), a non-contact air-puff tonometer which ejects an air impulse lasting 20 milliseconds and monitors the time course changes of the cornea by an electro-optical collimation detector system. Genotyping was carried out by using Illumina (San Diego, CA) genotyping platforms; the Human Hap 300k Duo and Human Hap610 Quad array. All SNPs passed quality control criteria (Hardy-Weinberg equilibrium p>0.001, minor allele frequency of at least 0.04, genotyping success rate for the SNP at least 95%). Imputation was calculated with reference to HapMap release 22 CEU by using IMPUTE version 2. Data from 2,235 participants, from 1,417 sibships/families (of which 209 monozygotic), were included in the analyses. For a subset of 2,093 participants from 1,331 sibships, also CCT data were available. The Goldmann-correlated IOP, which the manufacturers have calibrated with Goldmann applanation tonometry, was used as the outcome measure most comparable with the discovery cohort in this study. The mean IOP was calculated from 4 readings (2 from each eye) for each participant. Every association analysis was performed by using Merlin, given the family data. Zigocity was included in the model and was useful in modelling environmental variance.

*Australian Twin study*

The Australian Twin Eye Study comprises participants examined as part of the Twins Eye Study in Tasmania or the Brisbane Adolescent Twins Study. In most participants, the IOP was measured with the TONO-PEN XL (Reichert, Inc. New York, USA) as outlined in Mackey et al.^1^ The Australian cohorts were genotyped on the Illumina Human Hap610W Quad array, with part of the sample typed alongside the TwinsUK cohort and the remainder typed as a separate contract with DeCODE genetics. The inclusion criteria for the SNPs were a minor allele frequency >0.01, Hardy-Weinberg equilibrium p≥10^-6^, and a SNP call rate >95% or Illumina Beadstudio Gencall Score ≥0.7, resulting in 543,862 SNPs. Imputation was done with reference to HapMap release 22 CEU using MACH (<http://www.sph.umich.edu/csg/abecasis/MACH/>).^2^ Data from 1,807 people, from 863 families, were included in the analyses. The mean IOP of both eyes was used as outcome variable. Association analyses were performed in Merlin (<http://www.sph.umich.edu/csg/abecasis/merlin/>) by using the –fastassoc option. Age, sex and measurement technique (tonopen or Goldmann applanation tonometry) were fitted as covariates. Ancestry, initially determined through self-reporting, was verified through Principal Component decomposition.

*Diabetes Control and Complications Trial / Epidemiology of Diabetes Interventions and Complications study (DCCT/EDIC).*

The Diabetes Control and Complications Trial (DCCT, 1982-1993) is a multicenter, randomized clinical trial to compare conventional and intensive diabetic treatments in regard to their effects on the development and progression of long-term diabetic complications. The goal of intensive therapy was to normalize plasma glucose level. A total of 1,441 patients with type 1 diabetes were separated into two cohorts (primary prevention cohort and secondary intervention cohort) based on diabetes duration and presence of complications at baseline. The DCCT was prematurely stopped in 1993; after it was conclusively shown that intensive treatment delays the development and progression of long-term diabetic complications. Most of the DCCT subjects were further followed in the Epidemiology of Diabetes Intervention and Complications study (EDIC, 1994-present), an observational study to look at long-term effects of glycemic exposure. During the DCCT, participants went through annual ophthalmic exams. In each visit IOP was measured in both eyes by Goldmann applanation tonometry (on average 6 measurements). Genotyping was performed by using the Illumina 1M chip (San Diego, CA). After quality control measures, data of 841,342 SNPs with a minor allele frequency >0.01 were available. Genotypes for a total of 2.5M SNPs were imputed based on HapMap II CEU. After exclusion of any participants with a history of glaucoma, any prior eye surgery or ophthalmic medications, presence of angle neovascularization, as well as exclusion of any individuals who were likely to be admixed between white Europeans and other ethnic groups, data from 1,304 participants were included in the analyses.^3^ The mean IOP of both eyes was used as the outcome phenotype. To correct potential outliers, the top and bottom 0.5 percentiles of data were winsorized.

*Wellcome Trust Case-Control Consortium 2 (WTCCC2) / Blue Mountains Eye Study (BMES)*

Participants of the Wellcome Trust Case Control Consortium 2 (WTCCC2) are part of the Blue Mountains Eye Study (BMES), a population-based eye disease survey in individuals living in the Blue Mountains region, west of Sydney, Australia. The BMES protocol has been described in detail previously.^4^ Intraocular pressure (IOP) was measured by applanation tonometry using a Goldmann tonometer (Haag-Streit, Bern, Switzerland). Samples were genotyped on the Human660W-Quad. Imputation was performed with IMPUTE2^5^ which adopts a two-stage approach using both haploid and diploid reference panels. For the haploid reference panel, we used HapMap2 and HapMap3 SNP data for the 120 non-related CEU trios (see www.hapmap.org), and for the diploid reference we used the 1958 Birth Cohort (58C) and the United Kingdom Blood Service (UKBS) control data, merging genotypes from the Illumina 1.2M Duo chip and Affymetrix Genome Wide Human SNP array 6.0. Outlying individuals on the basis of call rate, heterozygosity, relatedness, ancestry, and signal intensity, or where there was discordance of reported gender and findings of gender specific markers, were excluded. The SNPs considered in this study passed the following quality control criteria in the WTCCC2/BMES data: minor allele frequency higher than 0.01, missing rate lower than 2%, Hardy Weinberg p > 1x10^-3^, Fisher information higher than 0.98 and no plate effect. Imputed SNPs had imputation information higher than 0.90. After exclusion of any participants who had undergone eye surgery, who were on medication designed to lower IOP or who had outlying values of IOP, data from 2136 individuals were considered in the analysis. The mean IOP of both eyes was considered as the response variable and the analysis was adjusted on age and sex. We performed single SNP analysis under an additive model using missing data likelihood score tests as implemented in SNPTEST.

**Glaucoma case-control studies**

Demographic and clinical characteristics of the glaucoma cases and controls of the 4 studies are presented in Table S4.

*Glaucoma case-control study in RS-I*

A total of 188 prevalent and incident glaucoma cases were recruited as part of RS-I. Glaucoma diagnosis was based on glaucomatous visual field loss. Cases were classified as glaucoma if the participant was classified as having glaucomatous visual field loss during at least one of the examination rounds. The visual field of each eye was screened by using a 52-point supra-threshold test that covered the central visual field with a radius of 24° (Humphrey Field Analyzer [HFA] II 740; Carl Zeiss, Oberkochen, Germany). The test was modified from a standard 76-point screening test and tested the same locations as used in the Glaucoma Hemifield Test.^6, 7^ If the first visual field test was unreliable, or a reliable test showed visual field loss in at least one eye, a second supra-threshold test was performed on that eye. In participants in which visual field loss remained present on the second supra-threshold test or the test was unreliable again, Goldmann kinetic perimetry (baseline and first follow-up; Haag-Streit, Bern, Switzerland) or full-threshold HFA testing with 24-2 grid (second follow-up visit) was performed on both eyes by a skilled perimetrist. The classification process of both Goldmann perimetry and full-threshold HFA test results has been described before.^6, 8^

*Genetic Research in Isolated Populations (GRIP) program.*

A total of 104 patients with glaucoma were recruited in three local hospitals in the region of the ERF population. These patients did not participate in the ERF study, which was used as control population. The diagnosis of glaucoma was made by the ophthalmologist in attendance and verified by a glaucoma specialist (HGL). The diagnosis was based on a glaucomatous appearance of the optic disc (notching or thinning of the neuroretinal rim), combined with a matching glaucomatous visual field defect and open angles on gonioscopy. Visual fields were tested with standard automated perimetry by means of the HFA 24-2 SITA Standard test program or the Octopus 101 (Haag Streit, Bern, Switzerland) G2 program with TOP strategy. Visual field test results had to be reliable and reproducible. Patients with any other known disease that could cause visual field defects were excluded. Genotyping was performed with the 318K array of the Illumina Infinium II whole-genome genotyping assay (HumanHap300-2). Genotyping quality control criteria and methods of imputations were identical to those in the ERF study.

*Amsterdam Glaucoma Study*

A total of 152 patients with glaucoma and 141 control persons were recruited from eye clinics, meetings of the glaucoma patients’ association, nursing homes, and fairs for the elderly. Preferably spouses of cases were used as control persons. If no spouse was available any non-related acquaintance was considered as suitable. In all persons, ophthalmoscopy and biomicroscopy with a 90 diopter lens were performed and digital stereo images of the optic nerve head were taken in mydriasis. Criteria for glaucoma included a glaucomatous optic neuropathy (vertical cup-disc ratio (VCDR) > 0.7) with corresponding glaucomatous visual field loss in at least one eye or a VCDR ≥ 0.8 when no visual field was available. Criteria for a control were age older than 60 years, and a VCDR ≤ 0.6 on fundus photography. Genotyping was performed by means of Taqman®.

*Glaucoma case-control studies Erlangen and Tübingen*

A total of 988 glaucoma cases and 378 healthy controls were recruited as part of case-control studies in Erlangen and Tübingen, Germany. Controls were age and gender matched to the patients. All participants underwent standardized clinical examinations for glaucoma at the Ophthalmology Department of the University of Erlangen-Nuremberg and at the University Eye Hospital in Würzburg and Tübingen, respectively. The examinations included optic nerve head imaging (Heidelberg Retina Tomograph [HRT] 1 and 2; or biomicroscopy with a Goldmann lens and a Haag-Streit slit lamp), visual field testing, and 24-hour Goldmann applanation tonometry profile with five measurements.^9, 10^ Glaucoma was defined as the presence of glaucomatous optic disc damage (as classified according to Jonas)^11, 12^ in at least one eye, with a corresponding visual field defect. A pathologic visual field was defined by a pathologic Bebie curve, three adjacent test points with more than 5 dB sensitivity loss, or at least one point with more than 15 dB sensitivity loss. Genotyping was preformed by means of selected pre-developed TaqMan® Genotyping Assays (Applied Biosystems, Foster City, CA, USA), following the manufacturer’s instructions.

**Additional results**

The data of the discovery cohorts were re-analyzed after exclusion of any participants who received IOP lowering treatment or who had received this treatment in the past (either by medication or surgically). For rs11656696[A], the effect on IOP in the final meta-analysis changed from -0.19 (SE=0.03; p=1.4x10^-8^) to -0.17 (SE=0.03; p=2.5x10^-7^) after exclusion of 139 participants from RS-I, 83 from RS-II, 33 from RS-33, and 23 from the ERF study. This change was mainly caused by a diminished effect of rs11656696[A] in RS-I. We therefore evaluated the genotypes of the participants of RS-I who received IOP lowering treatment, and found a significant reduction (p=8.1x10^-6^) in the frequency of the protective A-allele when compared to the RS-I population as a whole (odds ratio=0.58, 95%CI=0.45-0.74). This difference is consistent with the protective effect of rs11656696[A] on IOP and confirms that participants who receive IOP lowering treatment are less likely to carry the protective A-allele, and that exclusion of these participants merely results in a loss of statistical power. For rs7555523[C], the effect slightly diminished from 0.28 (SE=0.05; p=1.6x10^-8^) to 0.26 (SE=0.05; p=3.3x10^-8^).

Reference List

1. Mackey,D.A. *et al.* Twins eye study in Tasmania (TEST): rationale and methodology to recruit and examine twins. *Twin. Res. Hum. Genet.* **12**, 441-454 (2009).

2. Medland,S.E. *et al.* Common variants in the trichohyalin gene are associated with straight hair in Europeans. *Am. J. Hum. Genet.* **85**, 750-755 (2009).

3. Paterson,A.D. *et al.* A genome-wide association study identifies a novel major locus for glycemic control in type 1 diabetes, as measured by both A1C and glucose. *Diabetes* **59**, 539-549 (2010).

4. Mitchell,P., Smith,W., Attebo,K., & Healey,P.R. Prevalence of open-angle glaucoma in Australia. The Blue Mountains Eye Study. *Ophthalmology* **103**, 1661-1669 (1996).

5. Howie,B.N., Donnelly,P., & Marchini,J. A flexible and accurate genotype imputation method for the next generation of genome-wide association studies. *PLoS. Genet.* **5**, e1000529 (2009).

6. Skenduli-Bala,E. *et al.* Causes of incident visual field loss in a general elderly population: the Rotterdam study. *Arch. Ophthalmol.* **123**, 233-238 (2005).

7. Wolfs,R.C. *et al.* Changing views on open-angle glaucoma: definitions and prevalences--The Rotterdam Study. *Invest Ophthalmol. Vis. Sci.* **41**, 3309-3321 (2000).

8. Czudowska,M.A. *et al.* Incidence of glaucomatous visual field loss: a ten-year follow-up from the Rotterdam Study. *Ophthalmology* **117**, 1705-1712 (2010).

9. Pasutto,F. *et al.* Heterozygous NTF4 mutations impairing neurotrophin-4 signaling in patients with primary open-angle glaucoma. *Am. J. Hum. Genet.* **85**, 447-456 (2009).

10. Weisschuh,N., Wolf,C., Wissinger,B., & Gramer,E. Variations in the WDR36 gene in German patients with normal tension glaucoma. *Mol. Vis.* **13**, 724-729 (2007).

11. Jonas,J.B., Gusek,G.C., & Naumann,G.O. Optic disc morphometry in chronic primary open-angle glaucoma. I. Morphometric intrapapillary characteristics. *Graefes Arch. Clin. Exp. Ophthalmol.* **226**, 522-530 (1988).

12. Jonas,J.B. & Papastathopoulos,K. Ophthalmoscopic measurement of the optic disc. *Ophthalmology* **102**, 1102-1106 (1995).
